# Stout camphor tree genome fills gaps in understanding of flowering plant genome evolution

**DOI:** 10.1038/s41477-018-0337-0

**Published:** 2019-01-09

**Authors:** Shu-Miaw Chaw, Yu-Ching Liu, Yu-Wei Wu, Han-Yu Wang, Chan-Yi Ivy Lin, Chung-Shien Wu, Huei-Mien Ke, Lo-Yu Chang, Chih-Yao Hsu, Hui-Ting Yang, Edi Sudianto, Min-Hung Hsu, Kun-Pin Wu, Ling-Ni Wang, James H. Leebens-Mack, Isheng J. Tsai

**Affiliations:** 1 Biodiversity Research Center, Academia Sinica, Taipei, Taiwan; 2 Graduate Institute of Biomedical Informatics, College of Medical Science and Technology, Taipei Medical University, Taipei, Taiwan; 3 School of Medicine, National Taiwan University, Taipei, Taiwan; 4 Institute of Biomedical Informatics, National Yang-Ming University, Taipei, Taiwan; 5 Plant Biology Department, University of Georgia, Athens, GA USA

**Keywords:** Comparative genomics, Phylogenomics, Genome evolution, Plant evolution

## Abstract

We present reference-quality genome assembly and annotation for the stout camphor tree (*Cinnamomum kanehirae* (Laurales, Lauraceae)), the first sequenced member of the Magnoliidae comprising four orders (Laurales, Magnoliales, Canellales and Piperales) and over 9,000 species. Phylogenomic analysis of 13 representative seed plant genomes indicates that magnoliid and eudicot lineages share more recent common ancestry than monocots. Two whole-genome duplication events were inferred within the magnoliid lineage: one before divergence of Laurales and Magnoliales and the other within the Lauraceae. Small-scale segmental duplications and tandem duplications also contributed to innovation in the evolutionary history of *Cinnamomum*. For example, expansion of the terpenoid synthase gene subfamilies within the Laurales spawned the diversity of *Cinnamomum* monoterpenes and sesquiterpenes.

## Abstract (web summary)

A high-quality reference genome of the stout camphor tree reveals its genome evolution and supports that magnoliid and eudicot lineages share more common ancestry relative to monocots.

## Main

Aromatic medicinal plants have long been utilized as spices or curative agents throughout human history. In particular, many commercial essential oils are derived from flowering plants in the tree genus *Cinnamomum* L. (Lauraceae). For example, camphor, a bicyclic monoterpene ketone (C_10_H_16_O) that can be obtained from many members of this genus, has important industrial and pharmaceutical applications^1^. *Cinnamomum* includes approximately 250 species of evergreen aromatic trees belonging to Lauraceae (laurel family), which is an economically and ecologically important family that includes 2,850 species distributed mainly in tropical and subtropical regions of Asia and South America^2^. Among them, avocado (*Persea americana*), bay laurel (*Laurus nobilis*), camphor tree or camphor laurel (*Cinnamomum camphora*), cassia (*Cinnamomum cassia*) and cinnamon (including several *Cinnamomum* spp.) are important spice and fruit species. Lauraceae has traditionally been classified as one of the seven families of Laurales, which together with Canellales, Piperales and Magnoliales constitute the Magnoliidae (‘magnoliids’ informally).

The magnoliids (Magnoliidae), containing about 9,000 species, are characterized by three-merous flowers with diverse volatile secondary compounds, one-pored pollen and insect pollination^3^. Many magnoliids—such as custard apple (*Annonaceae*), nutmeg (*Myristica*), black pepper (*Piper nigrum*), magnolia and tulip tree (*Liriodendron tulipifera*)—produce economically important fruits, spices, essential oils, drugs, perfumes, timber and horticultural ornamentals. However, the phylogenetic position of magnoliids has been uncertain. They were considered to be (1) sister to the Chloranthaceae^4^, (2) sister to the monocots^5^, (3) sister to the clade containing monocots and eudicots^6^, (4) sister to the clade composing Chloranthaceae and Ceratophyllaceae^7^, or (5) sister to the clade including eudicots and Chloranthaceae–Ceratophyllaceae^8^, based on plastid genes, plastomic inverted repeat regions, four mitochondrial genes, inflorescence and floral structures, and 59 conserved nuclear genes, respectively. Similar to the Angiosperm Phylogeny Group (APG) III system, the APG IV system^9^ placed Magnoliidae and Chloranthaceae together as sister to a robust clade, including monocots and Ceratophyllales + eudicots. Furthermore, there are also unresolved questions about genome evolution within the Magnoliidae. Analysis of transcriptome sequences has implicated two rounds of genome duplication in the ancestry of *Persea* (Lauraceae) and one in the ancestry of *Liriodendron* (Magnoliaceae)^10^, but the relative timing of these events remains ambiguous.

*Cinnamomum kanehirae*, commonly known as the stout camphor tree (SCT), a name referring to its bulky, tall and strong trunk, is endemic to Taiwan and under threat of extinction. It has a restricted distribution in broadleaved forests in an elevational band between 450 and 1,200 m^11^. *Cinnamomum*, including SCT and six congeneric species, contributed to Taiwan’s position as the largest producer and exporter of camphor in the nineteenth century, and the value of their wood was further enhanced by their massive trunk diameters—the largest diameters among flowering plants of Taiwan—and their aromatic, decay-resistance quality that has been attributed to the essential oil d-terpinenol^12^. *Antrodia cinnamomea* is a parasitic fungus that infects the trunks of SCT causing heart rot^13^. The fungus produces several medicinal triterpenoids that impede the growth of liver cancer cells^14^ and act as antioxidants that protect against atherosclerosis^15^. Owing to intensive deforestation in the past half century, followed by poor seed germination and illegal logging to cultivate the fungus, natural populations of SCT are fragmented and threatened^16^.

Here, we report a chromosome-level genome assembly of SCT. Comparative analyses of the SCT genome with those of ten other angiosperms and two gymnosperms (ginkgo and Norway spruce) allow us to resolve the phylogenetic position of the magnoliids and shed new light on flowering plant genome evolution. Several gene families seem to be uniquely expanded in the SCT lineage, including the terpenoid synthase superfamily. Terpenoids play vital primary roles as photosynthetic pigments (carotenoids), electron carriers (plastoquinone and ubiquinone side chains) and regulators of plant growth (the phytohormone gibberellin and phytol side chain in chlorophyll)^17^. Specialized volatile or semi-volatile terpenoids are also important biological and ecological signals that protect plants against abiotic stress and promote beneficial biotic interactions above and below the ground with pollinators, pathogens, herbivorous insect and soil microorganisms^17^. Analyses of the SCT genome inform understanding of gene family evolution contributing to terpenoid biosynthesis, shed light on early events in flowering plant diversification and provide new insights into the demographic history of SCT with important implications for future conservation efforts.

## Results

### Assembly and annotation of *C.* *kanehirae*

SCT is diploid (2n = 24; Supplementary Fig. 1a) with an estimated genome size of 823.7 ± 58.2 Mb/1 C (Supplementary Figs. 1b and 2). We produced an assembly derived solely from 85× PacBio long reads (read N50 = 11.1 kb; contig N50 = 0.9 Mb) spanning 728.3 Mb. The consensus sequences of the assembly were corrected using 141× Illumina reads and further scaffolded with 207× ‘Chicago’ reconstituted chromatin and 204× Hi-C paired-end reads using the HiRise pipeline (Supplementary Fig. 3). A final, integrated assembly of 730.7 Mb was produced in 2,153 scaffolds, comprising 91.3% of the flow cytometry genome size estimate. The final scaffold N50 was 50.4 Mb with more than 90% in 12 pseudomolecules (Supplementary Table 1), presumably corresponding to the 12 SCT chromosomes.

Using a combination of reference plant protein homology support and transcriptome sequencing derived from various tissues (Supplementary Fig. 1c and Table 2) and ab initio gene prediction, 27,899 protein-coding gene models were annotated using the MAKER2 pipeline^18^ (Supplementary Table 1). Of these, 93.7% were found to be homologous to proteins in the TrEMBL database and 50% could be assigned Gene Ontology terms using eggNOG-mapper^19^. The proteome was estimated to be at least 89% complete based on BUSCO^20^ (benchmarking universal single-copy orthologs) assessment, which is comparable to other sequenced plant species (Supplementary Table 1). Orthofinder^21^ clustering of SCT gene models with those from 12 diverse seed plant genomes yielded 20,658 orthologous groups (Supplementary Table 3). 24,148 SCT genes (86.56%) were part of orthologous groups with orthologues from at least one other plant species. 3,744 gene models were not orthologous to others, and only 210 genes were part of the 48 SCT-specific orthologous groups. Altogether, they suggest that the phenotypic diversification in magnoliids may be fuelled by de novo birth of species-specific genes and expansion of existing gene families.

### Genome characterization

**Fig. 1:**
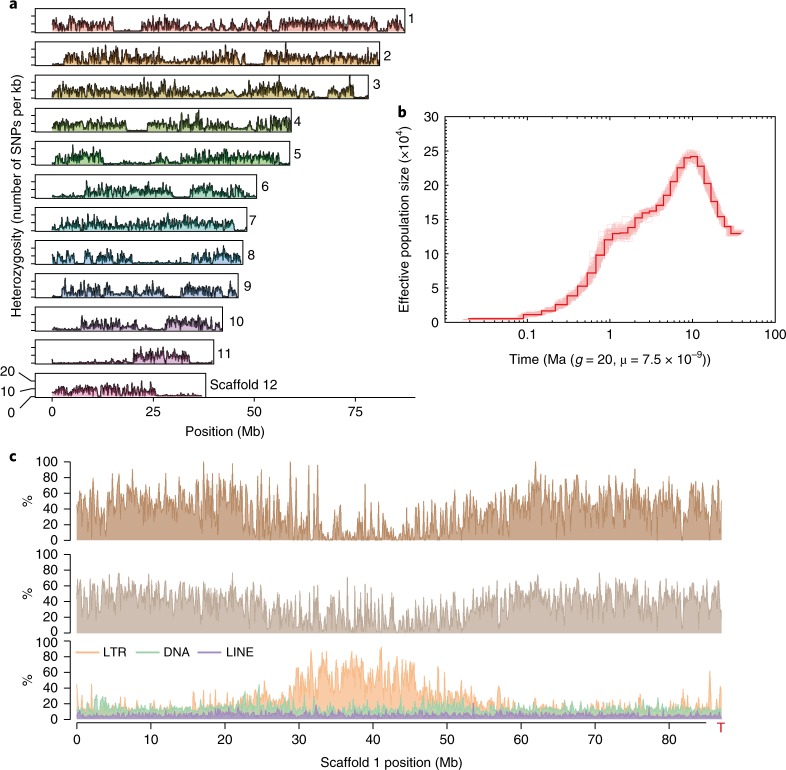
SCT genome heterozygosity. **a**, Number of heterozygous biallelic SNPs per 100-kb non-overlapping windows is plotted along the largest 12 scaffolds. Indels were excluded. **b**, The history of effective population size was inferred using the PSMC method. One hundred bootstraps were performed and the margins are shown in light red. **c**, For every non-overlapping 100-kb window, the distribution is shown from top to bottom: gene density (percentage of nucleotides with predicted model), transcriptome (percentage of nucleotides with evidence of transcriptome mapping) and three different classes of repetitive sequences (percentage of nucleotides with transposable element annotation). The red T letter denotes the presence of a telomeric repeat cluster at the scaffold end. LINE, long interspersed nuclear element.

We identified 3,950,027 biallelic heterozygous sites in the SCT genome, corresponding to an average heterozygosity of 0.54% (one heterozygous single nucleotide polymorphism (SNP) per 185 bp). The alternative (non-reference) allele frequencies at these sites had a major peak around 50% consistent with the fact that SCT is diploid with no evidence for recent aneuploidy (Supplementary Fig. 4). The spatial distribution of heterozygous sites was highly variable, with 23.9% of the genome exhibiting less than 1 SNP locus per kb compared to 10% of the genome with at least 12.6 SNP loci per kb. Runs of homozygosity regions appeared to be distributed randomly across SCT chromosomes, reaching a maximum of 20.2 Mb in scaffold 11 (Fig. 1a). Such long runs of homozygosity regions have equal sequence coverage than the rest of the genome (Supplementary Fig. 5) and may be associated with selective sweeps, inbreeding or recent population bottlenecks. Genes located in these runs of homozygosity regions were found to be enriched in lignin biosynthetic process and galactose metabolism (Supplementary Table 4), which suggest some potential roles in the formation of lignin–carbohydrate complexes^22^. Pairwise sequentially Markovian coalescent^23^ (PSMC) analysis based on heterozygous SNP densities implicated a continuous reduction of effective population size over the past 9 million years (Fig. 1b), with a possible bottleneck coincident with the mid-Pleistocene climatic shift 0.9 million years ago (Ma). Such patterns may reflect a complex population history of SCT associated with the geological history of Taiwan, including uplift and formation of the island in the late Miocene (9 Ma) followed by mountain building 5–6 Ma, respectively^24^.

Transposable elements and interspersed repeats made up 48% of the genome assembly (Supplementary Table 5). The majority of the transposable elements belonged to long terminal repeat (LTR) retrotransposons (25.53%), followed by DNA transposable elements (12.67%). Among the LTRs, 40.75% and 23.88% retrotransposons belonged to Ty3/Gypsy and Ty1/Copia, respectively (Supplementary Table 5). Phylogeny of the reverse transcriptase domain showed that the majority of Ty3/Gypsy copies formed a distinct clade (20,092 copies), presumably as a result of recent expansion and proliferation, whereas Ty1/Copia elements were grouped into two sister clades (7,229 and 2,950 copies) (Supplementary Fig. 6). With the exception of two scaffolds, both Ty3/Gypsy and Ty1/Copia LTR transposable elements were clustered within the pericentromeric centres of the 12 largest scaffolds (Fig. 1c and Supplementary Fig. 7). In addition, the LTR-enriched regions (defined by 100 kb with an excess of 50% comprising LTR class transposable elements) had on average 35% greater coverage than the rest of the genome (Fig. 1c and Supplementary Fig. 8), suggesting that these repeats were collapsed in the assembly and may have contributed to the differences in flow cytometry and k-mer genome size estimates. The coding sequence content of SCT is similar to the other angiosperm genomes included in our analyses (Supplementary Table 1), whereas introns are slightly longer in SCT owing to a higher density of transposable elements (*P* < 0.001, Wilcoxon rank-sum test; Supplementary Fig. 9).

As has been described for other plant genomes^25^, the chromosome-level scaffolds of SCT exhibit low protein-coding gene density and high transposable element density in the centres of chromosomes, and increased gene density towards the chromosome ends (Fig. 1c). We identified clusters of a putative subtelomere heptamer, TTTAGGG, extending as long as 2,547 copies, which implicate telomeric repeats in plants^26^ (Supplementary Table 6). In addition, 687 kb of nuclear plastid DNA-like sequences (NUPTs), averaging around 202.8 bp, were uncovered (Supplementary Table 7). SCT NUPTs were overwhelmingly dominated by short fragments, with 96% of the identified NUPTs less than 500 bp (Supplementary Table 8). The longest NUPT is ~20 kb in length and syntenic with 99.7% identity to a portion of the SCT plastome that contains seven protein-coding and five tRNA genes (Supplementary Fig. 10).

### Phylogenomic placement of *C.* *kanehirae* sister to eudicots

**Fig. 2:**
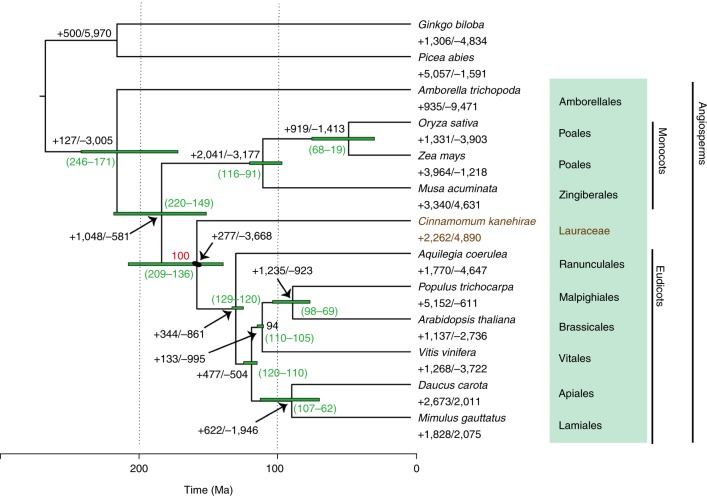
A species tree on the basis of 211 single-copy orthologues from 13 plant species. Gene family expansion and contraction are denoted in the numbers next to the plus and minus signs, respectively. The green numbers in the brackets denote divergence time estimates. All nodes’ bootstrap support was 100 unless stated otherwise.

To resolve the long-standing debate over the phylogenetic placement of magnoliids relative to other major flowering plant lineages, we constructed a phylogenetic tree based on 211 strictly single-copy orthologue sets (that is, one and only one homologue in all species) identified through OrthoFinder^21^ gene family circumscription of all gene models from the SCT and 12 other seed plant genomes (see Methods). A single species tree was recovered through maximum likelihood analysis^27^ of a concatenated supermatrix of the single-copy gene alignments and coalescent-based analysis using the 211 gene trees^28^ (Fig. 2 and Supplementary Fig. 11). SCT, representing the magnoliid lineage, was placed as sister to the eudicot clade (Fig. 2). This topology remained robust when we included a transcriptome data set of an additional 22 species of magnoliids order from the 1,000 plants initiative^29^ (1KP), although lower bootstrap support was obtained (Supplementary Fig. 12). Using MCMCtree^30^ with fossil calibrations, we calculated a 95% confidence interval for the time of divergence between magnoliids and eudicots to be 136.0–209.4 Ma (Fig. 2), which overlaps with two other recent estimates (114.8–164.1 Ma^31^ and 118.9–149.9 Ma^32^).

### Synteny analysis/whole-genome duplication

**Fig. 3:**
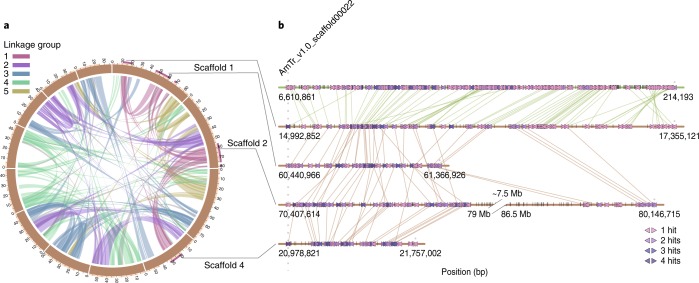
Evolutionary analysis of the SCT genome. **a**, Schematic representation of the intragenomic relationship among the 637 synteny blocks in the SCT genome. Synteny blocks (denoted by peach blocks) were assigned unambiguously into five linkage clusters representing ancient karyotypes and are colour coded. Purple blocks denote the synteny block assigned in the first linkage group (see also Supplementary Fig. 13). **b**, Schematic representation of the first linkage group within the SCT genome and their corresponding relationship in *A.* *trichopoda*.

Previous investigations of expressed sequence tags data inferred a genome-wide duplication within the magnoliids before the divergence of the Magnoliales and Laurales^10^, but synteny-based testing of this hypothesis has not been possible without an assembled magnoliid genome. A total of 16,498 gene pairs were identified in 992 syntenic blocks comprising 72.7% of the SCT genome assembly. Of these intragenomic syntenic blocks, 72.3% were found to be syntenic to more than one location on the genome, suggesting that more than one whole-genome duplication (WGD) occurred in the ancestry of SCT (Fig. 3a). Two rounds of ancient WGD were implicated by extensive synteny between pairs of chromosomal regions and significant but less syntenic pairing of each region with two additional genomic segments (Supplementary Fig. 13). Synteny blocks of SCT’s 12 largest scaffolds were assigned to five clusters that may correspond to pre-WGD ancestral chromosomes (Fig. 3a, Supplementary Fig. 13 and Supplementary Note).

*Amborella trichopoda* is the sole species representing the sister lineage to all other extant angiosperms and it has no evidence of WGD since divergence from the last common ancestor extant flowering plant lineages^33^. To confirm that two rounds of WGD took place in the ancestry of SCT after divergence of lineages leading to SCT and *A.* *trichopoda*, we assessed synteny between the two genomes. Consistent with our hypothesis, one to four segments of the SCT genome were aligned to a single region in the *A.* *trichopoda* genome (Fig. 3b and Supplementary Fig. 14).

**Fig. 4:**
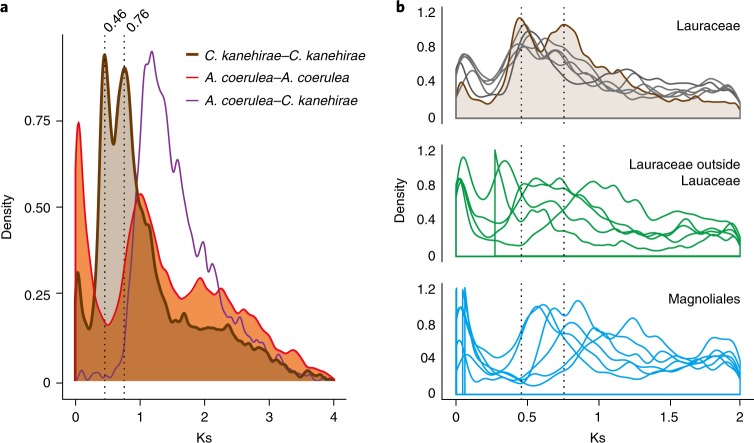
Density plots of synonymous substitutions (Ks) of the SCT genome and other plant species. **a**, Pairwise orthologue duplicates identified in synteny blocks within SCT, *A.* *coerulea* and between SCT and *A.* *coerulea*. **b**, Ks of intragenomic pairwise duplicates of the Lauraceae and the Magnoliales in the 1KP project^29^. Dashed lines denote the two Ks peaks observed in SCT. Brown and grey lines denote SCT and other Lauraceae’s Ks distribution, respectively.

To more precisely infer the timing of the two rounds of WGD evident in the SCT genome, intragenomic and interspecies homologue Ks (synonymous substitutions per synonymous site) distributions were estimated. SCT intragenomic duplicates showed two peaks around 0.46 and 0.76 (Fig. 4a), congruent with the two WGD events. Based on these two peaks, we were able to infer the karyotype evolution by organizing the clustered synteny blocks further into four groups presumably originating from one of the five pre-WGD chromosomes (Supplementary Fig. 15). Comparison between *Aquilegia coerulea* (Ranunculales, a sister lineage to all other extant eudicots^33^) and SCT orthologues revealed a prominent peak around Ks = 1.41 (Fig. 4a), whereas the *Aquilegia* intragenomic duplicate was around Ks = 1, implicating independent WGDs following the divergence of lineages leading to SCT and *Aquilegia*. The availability of the transcriptome of 17 Laurales + Magnoliales from the 1KP^29^ allowed us to test the hypothesized timing of the WGDs evident in the SCT genome^8^. Ks distribution of five out of six available species from Lauraceae revealed two peaks (Fig. 4b and Supplementary Fig. 16), as was seen in the SCT Ks distribution (Fig. 4a) and corresponding to two synteny-based inferences of WGDs in the ancestry of SCT (Fig. 3 and Supplementary Fig. 15). Only one Ks peak was observed in the remaining Laurales and Magnoliales species, suggesting only one WGD event occurred in the ancestry of these species (Supplementary Figs. 17 and 18). The Ks peak seen in *Aquilegia* data is probably attributable to WGD within the Ranunculales well after the divergence of eudicots and magnoliids (Fig. 4a).

### Specialization of the magnoliids proteome

We sought to identify genes and protein domains specific to SCT by annotating protein family (Pfam) domains and assessing their distribution across the 13 seed plant genomes included in our phylogenomic analyses. Consistent with the observation that there were very few SCT-specific orthologous groups, principal component analysis of Pfam domain content clustered SCT with the monocots and eudicots, with the first two principal components separating gymnosperms and *A.* *trichopoda* from this group (Supplementary Fig. 19a). There were considerable overlaps between SCT, eudicot and monocot species, suggesting significant functional diversification since these three lineages split. SCT also showed a significant enrichment and reduction of 111 and 34 protein domains compared to other plant species, respectively (Supplementary Fig. 19b and Supplementary Table 9). Gain of protein domains included the terpene synthase (TPS) carboxy-terminal domain involved in defence responses and the leucine-rich repeats (628 versus 334.4) in plant transpiration efficiency^34^. Interestingly, we found that SCT possesses 21 copies of EIN3/EIN3-like (EIL) transcription factor, more than the previously reported maximum of 17 copies in the banana genome (*Musa acuminata*)^35^. EILs initiate an ethylene signalling response by activating ethylene response factor (ERF), which we also found to be highly expanded in SCT (150 copies versus an average of 68.3 copies from nine species reported in ref. ^35^; Supplementary Fig. 20). ERF responds and positively modulates biosynthesis of phytohormonal signals, including ethylene^36^. Expression of ERF has been implicated in positively modulating plant development from fruit ripening^35^ to secondary growth in wood formation^37^, as well as in increased resistance to abiotic^38^ or biotic^39^ factors. Thus, expansion of EILs in SCT may stimulate ERF, leading to various regulation of downstream effectors that result in traits specific to SCT.

We next assessed orthologous group expansions and contractions across the seed plant phylogeny (Fig. 2). Gene family size evolution was dynamic across the phylogeny, and the branch leading to SCT did not exhibit significantly different numbers of expansions and contractions. Enrichment of Gene Ontology terms revealed either different gene families sharing common functions or single-gene families undergoing large expansions (Supplementary Tables 10 and 11). For example, expanded members of plant resistance (*R*) genes add up to ‘plant-type hypersensitive response’ (Supplementary Table 10). By contrast, the enriched Gene Ontology terms from the contracted gene families of the SCT branch (Supplementary Table 11) contain members of ABC transporters, indole-3-acetic acid-amido synthetase, xyloglucan endotransglucosylase/hydrolase and auxin-responsive protein, all of which are part of the ‘response to auxin’.

### *R* genes

The SCT genome annotation included 387 *R* gene models, 82% of which belong to nucleotide-binding site leucine-rich repeat (NBS-LRR) or coiled-coil NBS-LRR types. This result is consistent with a previous report that LRR is one of the most abundant protein domains in plants and it is highly likely that SCT is able to recognize and fight off pathogen products of avirulence (*Avr*) genes^40^. Among the sampled 13 genomes, SCT harbours the highest number of *R* genes among non-cultivated plants (Supplementary Fig. 21). The phylogenetic tree constructed from 2,465 NBS domains also suggests that clades within the gene family have diversified independently within the eudicots, monocots and magnoliids. Interestingly, the most diverse SCT NBS gene clades were sister to depauperate eudicot NBS gene clades (Supplementary Fig. 22).

### *TPS* gene family

**Fig. 5:**
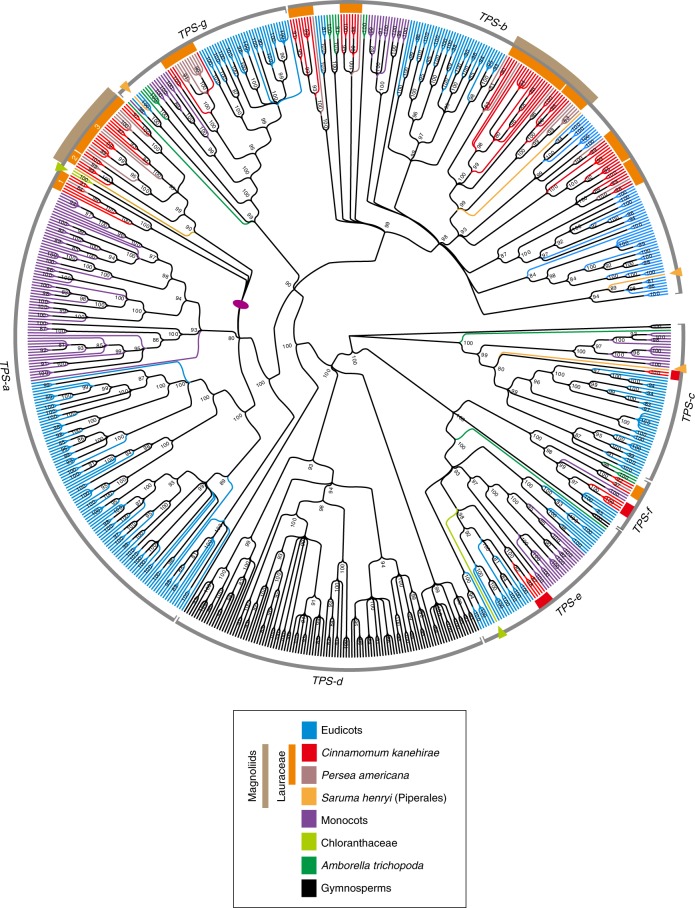
Phylogenetic placements of the 101 Ck*TPS* genes. The phylogenetic tree was constructed using putative or characterized *TPS* genes from 13 sequenced land plant genomes and two magnoliids with available transcriptomic data.

One of the most striking features of the SCT genome is the large number of *TPS* genes (Ck*TPS*). A total of 101 Ck*TPS* genes were predicted and annotated, the largest number for any other genome to date. By including a transcriptome data set of two more species from magnoliids (*P.* *americana* and *Saruma henryi*), phylogenetic analyses of *TPS* from 15 species placed Ck*TPS* genes among six of seven *TPS* gene subfamilies that have been described for seed plants^41^ (Fig. 5, Table 1 and Supplementary Figs. 23–28). Ck*TPS* genes placed in the *TPS-c* (2) and *TPS-e* (5) subfamilies probably encode diterpene synthases, such as copalyl diphosphate synthase and *ent*-kaurene synthase^42^. These are key enzymes catalysing the formation of the 20-carbon isoprenoids (collectively termed diterpenoids; C20s), which were thought to be eudicot specific^41^ and serve primary functions such as regulating plant primary metabolism. The remaining 94-predicted Ck*TPS* genes probably encode the 10-carbon monoterpene (C10) synthases, 15-carbon sesquiterpene (C15) synthases and additional 20-carbon diterpene (C20) synthases (Table 1). With 25 and 58 homologues, respectively, *TPS-a* and *TPS-b* subfamilies are most diverse in SCT, presumably contributing to the mass and mixed production of volatile C15s and C10s^43^.

**Table 1:** Numbers of *TPS* subfamilies in the 13 genomes and three transcriptomes of major seed plant lineages

		Primary metabolism		Secondary metabolism					
*TPS* subfamilies	Genome size (Mb)	c	e	a	b	d^a^	f	g	Total no.
Function species		CPS, C20	KS, C20	C15	IspS, C10	C10, C15, C20	C20	C10	
**Gymnosperms**									
*G.* *biloba*	10,609	1	1	–	–	49	–	–	51
*P.* *abies*	12,301	2	1	–	–	59	–	–	62
**Angiosperms**									
*A.* *trichopoda*	706	1	1	–	7	–	3	5	17
**Chloranthaceae**									
*S.* *glabra*^b^	–	–	1	2	–	–	–	–	3
**Magnoliids**									
**Lauraceae**									
*C.* *kanehirae*	731	2	5	25	58	–	7	4	101
*P.* *americana*^b^	–	–	–	11	12	–	1	9	33
**Piperales**									
*S.* *henryi*^b^	–	1	–	1	2	–	–	1	5
**Monocots**									
*M.* *acuminata*	473	2	2	21	13	–	3	3	44
*O.* *sativa*	375	3	10	19	–	–	–	1	33
*Z.* *mays*	2,068	8	6	30	2	–	–	5	51
**Eudicots**									
*A.* *coerulea*	307	15	13	12	34	–	–	8	82
**Rosids**									
*A.* *thaliana*	120	1	1	23	5	–	1	1	32
*P.* *trichocarpa*	473	2	2	16	14	–	1	3	38
*V.* *vinifera*^c^	434	2	1	29	10	–	2^c^	14	58
**Asterids**									
*D.* *carota*	422	3	2	1	15	–	1	7	29
*M.* *guttatus*	313	13	13	19	17	–	–	1	63

^a^*TPS-d* subfamily is gymnosperm specific. ^b^Transcriptome data of these three taxa were highly likely incomplete for covering all *TPS* transcripts, so that their total numbers of *TPS* were not reliable but are for reference only. ^c^These two *TPS-f* were previously characterized from grape floral cDNA without identical genomic Vv*TPS* genes (Martin et al.^103^); Vv*TPS* sequences labelled as unknown (Martin et al.^103^) in the *TPS* gene tree were not counted. CPS, copalyl diphosphate synthase; KS, kaurene synthase; IspS, isoprene synthase.

It is noteworthy that the *TPS* gene tree resolved Lauraceae-specific *TPS* gene clades within the *TPS-a*, *TPS-b*, *TPS-f* and *TPS-g* gene subfamilies (Supplementary Figs. 23, 24, 27 and 28). This pattern of *TPS* gene duplication in a common ancestor of *Persea* and *Cinnamomum* and subsequent retention may indicate subfunctionalization or neofunctionalization of duplicated *TPS* genes within the Lauraceae. A magnoliids-specific subclade in the *TPS-a* subfamily was also identified in analyses, including more magnoliid *TPS* genes with characterized functions (Supplementary Fig. 23). Indeed, we detected positive selection in the Lauraceae-specific *TPS-f* -I and -II subclades, implying functional divergence (Supplementary Table 13). Together, these data indicate increasing diversification of magnoliid *TPS* genes both before and after the origin of the Lauraceae.

Ck*TPS* genes are not uniformly distributed throughout the chromosomes (Supplementary Table 12) and clustering of members from individual subfamilies was observed as tandem duplicates (Supplementary Fig. 29). Seventy-six *TPS* genes were observed in the largest 12 scaffolds of SCT. Of those, 60.5% (46 copies) belonging to different subfamilies were found in the 0.5–15 Mb and 22.0–24.5 Mb region of scaffolds 7 and 10, respectively (Supplementary Fig. 29). Scaffold 7 contains 29 Ck*TPS* genes belonging to several subfamilies, including all of the eight Ck*TPS-a*, 12 Ck*TPS-b*, five Ck*TPS-e* and three Ck*TPS-f* (Supplementary Fig. 29). By contrast, only two members of Ck*TPS-c* reside in scaffold 1. Twenty-four Ck*TPS* genes are located in other smaller scaffolds, 22 of which encode the subfamily TPS-b (Supplementary Fig. 24). Some of these subfamilies located on scaffolds 7 and 10 are physically in proximity of each other (Supplementary Fig. 29). For instance, 3 out of 11 TPS-b-Lau III subfamily members were located adjacent to 4 out of 11 TPS-b-Lau V subfamily (Supplementary Fig. 29), whereas other subfamily members were found not in corresponding syntenic regions but elsewhere in the genome (Supplementary Fig. 30). Genes belonging to this cluster were not grouped together in their corresponding subfamily phylogeny (Supplementary Fig. 30), suggesting that their arrangement might have occurred more recently than the last WGD event.

## Discussion

It is currently challenging to find wild SCT populations, making the conservation and basic study of this tree a priority. Camphor trees have been intensively logged since the nineteenth century, initially for hardwood properties and association with the fungus *A.* *cinnamomea*. The apparent runs of homozygosity have been observed due to anthropogenic selective pressures or inbreeding in several livestock^44^, although inbreeding as a result of recent population bottleneck may be a more likely explanation for SCT. Interestingly, continuous decline in effective population size was inferred since 9 Ma. These observations may reflect a complex population history of SCT and Taiwan itself after origination and mountain building of the island that occurred around late Miocene (9 Ma) and 5–6 Ma, respectively^24^. The availability of the SCT genome will help the development of precise genetic monitoring and tree management for the survival of SCT’s natural populations.

Our phylogenomic analyses of 211 single-copy orthologues from 13 representative seed plant genomes, including the first magnoliid representative, SCT, resolve magnoliids to be closer to eudicots than to monocots. This result disagrees with APG IV’s resolution placing magnloliids as an outgroup to a clade containing monocots, Ceratophyllales and eudicots, but is in good agreement with a recent analysis of 59 orthologous nuclear genes based on transcriptome data of 26 seed plants^8^. Unfortunately, no complete genomic data of either Chloranthaceae or Ceratophyllacae are currently available for further re-examining the relationships of these two taxa, magnoliids, monocots, eudicots and the Amborella–Nymphaeles–Austrobaileyales grade. However, the placement of SCT as a sister to the eudicots in our analysis has important implications for comparative genomic analyses of evolutionary innovations within the eudicots, which comprise ~75% of extant flowering plants^8^. Consistent to early isozyme analysis^45^, within the Lauraceae, we identified the timing of two rounds of independent WGD events that contributed to gene family expansions and innovations in pathogen, herbivore and mutualistic interactions. Large Ks peak ranges in the Laurales and Magnoliales from the 1KP transcriptome data set may be due to variation of synonymous substitution rates in the different lineages^29^. Complete genome assemblies for representatives of additional magnoliid lineages are needed to pinpoint the exact timing of these WGD events. The SCT genome will serve as an important reference outgroup for reconstructing the timing and nature of polyploidy events that gave rise to the hexaploid ancestor of all core eudicots (Pentapetalae)^46,47^.

Gene tree topologies for each of the six angiosperm *TPS* subfamilies revealed diversification of *TPS* genes and gene function in the ancestry of SCT. The C20s, producing *TPS-f* genes, were suggested to be eudicot specific because both rice and sorghum lack this subfamily^41^. Our data clearly indicate that this subfamily was present in the last common ancestor of all angiosperms but was lost from the grass family (Table 1). Massive diversification of the *TPS-a* and *TPS-b* subfamilies within the Lauraceae is consistent with a previous report that the main constituents of 58 essential oils produced in *Cinnamomum* leaves are C10s and C15s^43^. These findings are in congruent with the fact that fruiting bodies of the SCT-specific parasitic fungus *Antrodia cinamomea* can produce 78 kinds of terpenoids, including 31 structure-different triterpenoids (C30s)^48^, many of which are synthesized via the mevalonate pathway, as are C10s and C15s followed by cyclizing squalenes (C_30_H_50_) into the skeletons of C30s^49^. It is reasonable to suggest that this fungus obtained intermediate compounds through decomposing trunk matters from SCT.

The 101 Ck*TPS* genes identified in the SCT genome are unevenly distributed across the 12 chromosomal scaffolds and include gene clusters from multiple subfamilies (Supplementary Fig. 30). In the *Drosophila melanogaster* genome, ‘tandem duplicate overactivity’ has been observed, with tandemly duplicated *Adh* genes showing 2.6-fold greater expression than single-copy *Adh* genes^50^. These rearrangement events may have also contributed to diversification of TPS enzymes in the SCT lineage and subsequent clustering of genes associated with mass production of terpenoids.

In summary, the availability of the SCT genome establishes a valuable genomic foundation that will help to unravel the genetic diversity and evolution of other magnoliids, and to give a better understanding of flowering plant genome evolution and diversification. At the same time, the reference-quality SCT genome sequence will enable efforts to conserve genome-wide genetic diversity in this culturally and economically important broadleaved forest species.

## Methods

### Plant materials

All plant materials used in this study were collected from a 12-year-old SCT growing in Ershui Township, Changhua County, Taiwan (23° 49′ 25.9″ N, 120° 36′ 41.2″ E) during April–July of 2014–2016. The tree was grown up from a seedling obtained from the Forestry Management Section, Department of Agriculture, Taoyuan City, Taiwan. The specimen (voucher number: Chaw 1501) was deposited in the Herbarium of Biodiversity Research Center, Academia Sinica, Taipei, Taiwan.

### Genomic DNA extraction and sequencing

We used a modified high-salt method^51^ to eliminate the high content of polysaccharides in SCT leaves, followed by total DNA extraction with a modified cetyltrimethylammonium bromide (CTAB) method^52^. Three approaches were employed in DNA sequencing. First, paired-end and mate-pair libraries were constructed using the Illumina TruSeq DNA HT Sample Prep Kit and Illumina Nextera Mate Pair Sample Prep Kit following the kit’s instructions, respectively. All obtained libraries were sequenced on an Illumina NextSeq 500 platform to generate ~278.8 Gb of raw data. Second, SMRT libraries were constructed using the PacBio 20-kb protocol (https://www.pacb.com/). After loading on SMRT cells (SMRT Cell 8Pac), these libraries were sequenced on a PacBio RS-II instrument using P6 polymerase and C4 sequencing reagent (Pacific Biosciences). Third, a Chicago and a Hi-C library were prepared by Dovetail Genomics (Santa Cruz) and sequenced on an Illumina HiSeq 2500 to generate 150-bp read pairs.

### RNA extraction and sequencing

Opening flowers, flower buds (two stages), immature leaves, young leaves, mature leaves, young stems and fruits were collected from the same individual (Supplementary Fig. 1c) and their total RNAs were extracted^53^. The extracted RNA was purified using poly-T oligo-attached magnetic beads. All transcriptome libraries were constructed using the Illumina TruSeq library Stranded mRNA Prep Kit and sequenced on an Illumina HiSeq 2000 platform. A summary of transcriptome data is shown in Supplementary Table 2.

### Chromosome number assessment

Root tips from cutting seedlings were used to examine the chromosome number based on Suen et al.’s method^54^. The stained samples were observed under a Nikon Eclipse 90i microscope (Supplementary Fig. 1a).

### Genome size estimation

Fresh leaves of SCT were finely chopped with a new razor blade in 250 µl isolation buffer (200 mM Tris, 4 mM MgCl_2_-6H_2_O and 0.5% Triton X-100) and mixed well, following the protocol of Dolezel et al.^55^. The mixture was filtered through a 40-μm nylon mesh, followed by incubation of the filtered suspensions with a DNA fluorochrome (50 μg ml^−1^ propidium iodide containing RNase A). Samples were analysed on the MoFlo XDP Cell Sorter (Beckman Coulter Life Science) and the Attune NxT Flow Cytometer (Thermo Fisher Scientific) in the Institute of Plant and Microbial Biology Flow Cytometry Analysis and Sorting Services at Academia Sinica, Taipei, Taiwan. Two and one replicates were performed on the former and latter machines, respectively, using chicken erythrocyte (BioSure) as an internal reference (Supplementary Fig. 1b). The 1 C genome size for SCT was estimated to be 781–890 Mb (Supplementary Figs. 1b and 2). Estimates of genome size from Illumina paired-end sequences were inferred using Genomescope^56^ (version 1.0; based on k-mer 31).

### De novo assembly of SCT

PacBio reads were assembled using the FALCON^57^ (version 0.5.0) assembler. The consensus sequences of the assembly were further corrected using PacBio reads using Quiver^58^ and Illumina reads using Pilon^59^ (version 1.22). The PacBio assembly was scaffolded using the HiRISE scaffolder^60^ (version July2015_GR), and consensus sequences were further improved using Pilon with one iteration^59^. The genome completeness was assessed using a plant data set of BUSCO^20^ (version 3.0.2). To identify putative telomeric repeats, the assembly was searched for high copy number repeats less than 10 bp using tandem repeat finder^61^ (version 4.09; options: 2 7 7 80 10 50 500). The heptamer TTTAGGG was identified (Supplementary Table 6).

### Gene predictions and functional annotation

Transcriptome paired-end reads were aligned to the genome using STAR^62^ (version 2.5.3a). Transcripts were identified using two approaches: (1) assembled de novo using Trinity^63^ (version 2.3.2) and (2) reconstructed using Stringtie^64^ (version 1.3.1c) as well as CLASS2 (ref. ^65^) (version 2.1.7). Transcripts generated from Trinity were remapped to the reference using GMAP^66^. The three sets of transcripts were merged and filtered using MIKADO^67^ (version 1.1). Proteomes from representative reference species (Uniprot plants; proteomes of *Amborella trichopoda* and *Arabidopsis thaliana*) were downloaded from Phytozome (version 12.1; https://phytozome.jgi.doe.gov/). The gene predictor Augustus^68^ (version 3.2.1) and SNAP^69^ were trained either on the gene models predicted using BRAKER1 (ref. ^70^) or MAKER2 (ref. ^18^) (version 2.31.9). The assembled transcripts, reference proteomes, BRAKER1 and the BUSCO predictions were combined as evidence hints for input of the MAKER2 (ref. ^18^) annotation pipeline. MAKER2 (ref. ^18^) invoked the two trained gene predictors to generate a final set of gene annotation. Amino acid sequences of the proteome were functionally annotated using Blast2GO^71^ and eggNOG-mapper^19^ (version 1.0.3). NUPTs of SCT were searched against its plastid genome (plastome; KR014245 (ref. ^72^)) using blastn (parameters were followed from Smith et al.^73^).

### Analysis of genome heterozygosity

Paired-end Illumina reads of SCT were aligned to reference using bwa mem^74^ (version 0.7.17-r1188). PCR duplicates were removed using SAMtools^75^ (version 1.8). Heterozygous biallelic SNPs were called using SAMtools^75^ and consensus sequences were generated using bcftools^76^ (version 1.7). Depth of coverage and alternative allele frequency plots were conducted using R version 3.4.2. The consensus sequence was fed to the PSMC program^23^ to infer past effective population size. All of the parameters used for the PSMC program were at default with the exception of -u 7.5 × 10^−9^ taken from *A.* *thaliana*^77^ and -g 20 taken from *Neolitsea sericea* (Lauraceae)^78^.

### Identification of repetitive elements

Repetitive elements were first identified by modelling the repeats using RepeatModeler^79^ and then searched and quantified repeats using RepeatMasker^80^. Repeat types modelled as ‘unknown’ by RepeatModeler were further annotated using TEclass^81^. Tandem repeats were identified using Tandem Repeats Finder^61^. The proportions of different types of repeats were quantified by dissecting the 12 largest scaffolds into 100,000-bp chunks and calculating the total lengths and percentages of the repetitive elements within the chunks. LTR retrotransposons (LTR-RT) domains were extracted following Guan et al.’s method^82^. Briefly, a two-step procedure was applied on the genomes. The first was to find candidate LTR-RTs similar to known reverse transcriptase domains and the second was to identify other LTR-RTs using the candidates identified in the first step. The identified LTR-RT domains were integrated with those downloaded from the Ty1/Copia and Ty3/Gypsy trees of Guan et al.^82^. Trees were built by aligning the sequences using MAFFT^83^ (version 7.310; --genafpair --ep 0) and applied FastTree^84^ with the Jones, Taylor and Thornton (JTT) model on the aligned sequences, and were coloured using the APE package^85^.

### Gene family or orthogroup inference and analysis of protein domains

The amino acid and nucleotide sequences of 12 representative plant species were downloaded from various sources: *A.* *coerulea*, *A.* *thaliana*, *Daucus carota*, *Mimulus guttatus*, *M.* *acuminata*, *Oryza sativa japonica*, *Populus trichocarpa*, *Vitis vinifera* and *Zea mays* from Phytozome (version 12.1; https://phytozome.jgi.doe.gov/), *Picea abies* from the Plant Genome Integrative Explorer Resource^86^ (http://plantgenie.org/), *Ginkgo biloba* from GigaDB^87^ and *A.* *trichopoda* from Ensembl plants^88^ (release 39). Gene families or orthologous groups of these species and SCT were determined by OrthoFinder^21^ (version 2.2.0). Pfams of each species were calculated from the Pfam website (version 31.0; https://pfam.xfam.org/). Pfam numbers of every species were transformed into z-scores. Significant expansions or reductions of Pfams in SCT were based on a z-score greater than 1.96 or less than −1.96, respectively. The significant Pfams were sorted by Pfam numbers (Supplementary Fig. 19). Gene family expansion and loss were inferred using CAFE^89^ (version 4.1, with an input tree as the species tree inferred from the single-copy orthologues).

### Phylogenetic analysis

MAFFT^83^ (version 7.271; option --maxiterate 1000) was used to align 13 sets of amino acid sequences of 211 single-copy orthologous groups. Each orthologous group alignment was used to compute a maximum likelihood phylogeny using RAxML^27^ (version 8.2.11; options: -m PROTGAMMAILGF -f a) with 500 bootstrap replicates. The best phylogeny and bootstrap replicates for each gene were used to infer a consensus species tree using ASTRAL-III^28^. A maximum likelihood phylogeny was constructed with the concatenated amino acid alignments of the single-copy orthogroups (version 8.2.11; options: -m PROTGAMMAILGF -f a), also with 500 bootstrap replicates.

### Estimation of divergence time

Divergence time of each tree node was inferred using MCMCtree of the PAML^30^ package (version 4.9g; options: correlated molecular clock, JC69 model and rest being default). The final species tree and the concatenated translated nucleotide alignments of 211 single-copy orthologues were used as input of MCMCtree. The phylogeny was calibrated using various fossil records or molecular divergence estimate by placing soft bounds at split node of:(1) *A.* *thaliana–V.* *vinifera* (115–105 Ma)^90^, (2) *M.* *acuminata–Z.* *mays* (115–90 Ma)^90^, (3) Ranunculales (128.63–119.6 Ma)^32^, (4) Angiospermae (247.2–125 Ma)^32^, (5) Acrogymnospermae (365.629–308.14 Ma)^32^ and (6) a hard bound of 420 Ma of outgroup *Physcomitrella patens*^91^.

### Analysis of genome synteny and WGD

Dot plots between SCT and *A.* *trichopoda* assemblies were produced using SynMap from the Comparative Genomics Platform (Coge^92^) to visualize the paleoploidy level of SCT. Synteny blocks within SCT and between *A.* *trichopoda* and *A.* *coerulea* were identified using DAGchainer^93^ (same parameters as Coge:^92^ -E 0.05 -D 20 -g 10 -A 5). Ks between syntenic group pairs were calculated using the DECIPHER^94^ package in R. Depth of the inferred syntenic blocks were calculated using Bedtools^95^. Both the Ks distribution and the syntenic block depth were used to determine the paleopolyploidy level^96^ of SCT. Using the quadruplicate or triplicate orthologues in the syntenic blocks as backbones, as well as *A.* *trichopoda* regions showing up to four syntenic regions, we identified the start and end coordinates of linkage clusters (Supplementary Note).

### *R* genes

*R* genes were identified based on ref. ^97^. Briefly, the predicted genes of the 13 sampled species were searched for the Pfam NBS (NB-ARC) protein family (PF00931) using HMMER version 3.1b2 (ref. ^98^) with an *e*-value cut-off of 1 × 10^−5^. Extracted sequences were then checked for protein domains using InterproScan^99^ (version 5.19–58.0) to remove false-positive NB-ARC domain hits. The NBS domains of the genes that passed both HMMER and InterproScan were extracted according to the InterproScan annotation and aligned using MAFFT^83^ (version 7.310; --genafpair --ep 0); the alignment was then input into FastTree^84^ with the JTT model and visualized using EvolView^100^.

### *TPS* genes

In addition to the 13 species proteome data set used in this study, transcriptome data from one Chloranthaceae species, *Sarcandra glabra*, and two magnollids representatives, *P.* *americana* (avocado) and *S.* *henryi* (saruma), were downloaded from the 1KP transcriptome database^29^. Previously annotated *TPS* genes of four species: *A.* *thaliana*^101^, *O.* *sativa*^41^, *P.* *trichocarpa*^102^ and *V.* *vinifera*^103^ were retrieved. For species without a priori *TPS* annotations, two Pfam domains: PF03936 and PF01397, were used to identify against the proteomes using HMMER^104^ (version 3.0; cut-off at *e* < 10^−5^). Pseudogenes and sequence lengths shorter than 200 amino acids were excluded from further analysis. Putative or annotated protein sequences of TPS (*n* = 702) were aligned using MAFFT^83^ (version 7.310 with default parameters) and manually adjusted using MEGA^105^ (version 7.0). The *TPS* gene tree was constructed using FastTree^84^ (version 2.1.0) with 1,000 bootstrap replicates. The subfamily *TPS-c* was designated as the outgroup. Branching nodes with bootstrap values of <80% were treated as collapsed.

### Reporting Summary

Further information on research design is available in the Nature Research Reporting Summary linked to this article.

## Supplementary information

Supplementary Information Supplementary Note; Supplementary Figures 1–30; and Supplementary Tables 1, 2, 5–8, 12 and 13.

Reporting Summary

Supplementary Tables Supplementary Tables 3, 4 and 9–11.

## Data Availability

All of the raw sequence reads used in this study have been deposited in NCBI under the BioProject accession number PRJNA477266. The assembly of SCT is available under the accession number GCA_003546025.1.
