# Supplementary material for: Stout camphor tree genome fills gaps in understanding of flowering plant genome evolution
Source: Nat Plants. 2019 Jan 9;5(1):63–73. doi: 10.1038/s41477-018-0337-0 (PMC6784883; doi:10.1038/s41477-018-0337-0)
Supplement: Supplementary file 1 — Supplementary Note; Supplementary Figures 1–30; and Supplementary Tables 1, 2, 5–8, 12 and 13. [file 41477_2018_337_MOESM1_ESM.pdf]

nature plants

SUPPLEMENTARY INFORMATION
https://doi.org/10.1038/s41477-018-0337-0

In the format provided by the authors and unedited.

Stout camphor tree genome fills gaps in understanding of flowering plant genome evolution

Shu-Miaw Chaw[1,6]*, Yu-Ching Liu[1], Yu-Wei Wu[2], Han-Yu Wang[1], Chan-Yi Ivy Lin[1], Chung-Shien Wu[1], Huei-Mien Ke[1], Lo-Yu Chang[1,3], Chih-Yao Hsu[1], Hui-Ting Yang[1], Edi Sudianto[1], Min-Hung Hsu[1,4], Kun-Pin Wu[4], Ling-Ni Wang[1], James H. Leebens-Mack[5] and Isheng J. Tsai[1,6]*

[1]Biodiversity Research Center, Academia Sinica, Taipei, Taiwan. [2]Graduate Institute of Biomedical Informatics, College of Medical Science and Technology, Taipei Medical University, Taipei, Taiwan. [3]School of Medicine, National Taiwan University, Taipei, Taiwan. [4]Institute of Biomedical Informatics, National Yang-Ming University, Taipei, Taiwan. [5]Plant Biology Department, University of Georgia, Athens, GA, USA. [6]These authors contributed equally: Shu-Miaw Chaw, Isheng J. Tsai. *e-mail: smchaw@sinica.edu.tw; ijtsai@sinica.edu.tw**Open Access** This article is licensed under a Creative Commons Attribution 4.0 International License, which permits use, sharing, adaptation, distribution and reproduction in any medium or format, as long as you give appropriate credit to the original author(s) and the source, provide a link to the Creative Commons license, and indicate if changes were made.
The images or other third party material in this article are included in the article's
Creative Commons license, unless indicated otherwise in a credit line to the material.
If material is not included in the article's Creative Commons license and your intended use is not permitted by statutory regulation or exceeds the permitted use, you will need to obtain permission directly from the copyright holder. To view a copy of this license, visit http://creativecommons.org/licenses/by/4.0/.

Supplementary Information

Stout camphor tree genome fills gaps in understanding of flowering plant genome evolution

Shu-Miaw Chaw, Yu-Ching Liu, Yu-Wei Wu, Han-Yu Wang, Chan-Yi Ivy Lin, Chung-Shien Wu, Huei-Mien Ke, Lo-Yu Chang, Chih-Yao Hsu Hui-Ting Yang, Edi Sudianto, Ming-Hung Hsu, Kun-Pin Wu, Ling-Ni Wang, Jim Leebens-Mack and Isheng. J. Tsai

I. SUPPLEMENTARY NOTE 3

Assignment of intragenomic synteny blocks into linkage clusters 3

II. SUPPLEMENTARY FIGURES..4

Supplementary Fig. 1 4
Supplementary Fig. 2 5
Supplementary Fig. 3 6
Supplementary Fig. 4 7
Supplementary Fig. 5 8
Supplementary Fig. 6 9
Supplementary Fig. 7 10
Supplementary Fig. 8 11
Supplementary Fig. 9 12
Supplementary Fig. 10 13
Supplementary Fig. 11 14
Supplementary Fig. 12 15
Supplementary Fig. 13 16
Supplementary Fig. 14 17
Supplementary Fig. 15 18
Supplementary Fig. 16 19
Supplementary Fig. 17 20
Supplementary Fig. 18 21
Supplementary Fig. 19 22
Supplementary Fig. 20 23

Supplementary Fig. 21 ……………………………………………………24
Supplementary Fig. 22 ……………………………………………………25
Supplementary Fig. 23 ……………………………………………………27
Supplementary Fig. 24 ……………………………………………………29
Supplementary Fig. 25 ……………………………………………………30
Supplementary Fig. 26 ……………………………………………………31
Supplementary Fig. 27 ……………………………………………………32
Supplementary Fig. 28 ……………………………………………………33
Supplementary Fig. 29 ……………………………………………………35
Supplementary Fig. 30 ……………………………………………………36

III. SUPPLEMENTARY TABLES……………………………………37

Supplementary Table 1 ……………………………………………………37
Supplementary Table 2 ……………………………………………………38
Supplementary Table 3 ……………………………………………………39
Supplementary Table 4 ……………………………………………………39
Supplementary Table 5 ……………………………………………………39
Supplementary Table 6 ……………………………………………………40
Supplementary Table 7 ……………………………………………………41
Supplementary Table 8 ……………………………………………………41
Supplementary Table 9 ……………………………………………………42
Supplementary Table 10 …………………………………………………42
Supplementary Table 11 …………………………………………………42
Supplementary Table 12 …………………………………………………43
Supplementary Table 13 …………………………………………………44

I. SUPPLEMENTARY NOTE

Assignment of intragenomic synteny blocks into linkage clusters

If a genome underwent two rounds of WGD, an ancestral gene may give rise to three paralogous copies and a gene cluster may be syntenic to three other regions. Under this rationale, we categorized all duplicated, triplicated and quadruplicated orthologous genes and regions within syntenic blocks as possible signals WGD. Only syntenic blocks with more than ten gene pairs defined by DAGchainer[1] was included in this analysis. Synteny blocks that are adjacent to each other in the assembly were merged using Bedtools[2] and custom python scripts if their corresponding matches were also adjacent to each other. Regions with more than four matches were not considered from this merging process. This resulted 338 synteny blocks with no gap into 81 areas on the longest 12 scaffolds. The merged blocks were then unambiguously classified into linkage clusters by linking the quadruplicated and triplicated orthologues between regions. We repeated this process iteratively to assign the unconnected synteny blocks in proximity to these clusters. Based on these criteria, 55.6% of synteny blocks consisting of 48.0% of the assembly were unambiguously assigned into either group (Supplementary Fig. 13), whilst 36 blocks with the cumulative length of 120.8 Mb were visually inspected and assigned.

II. SUPPLEMENTARY FIGURES

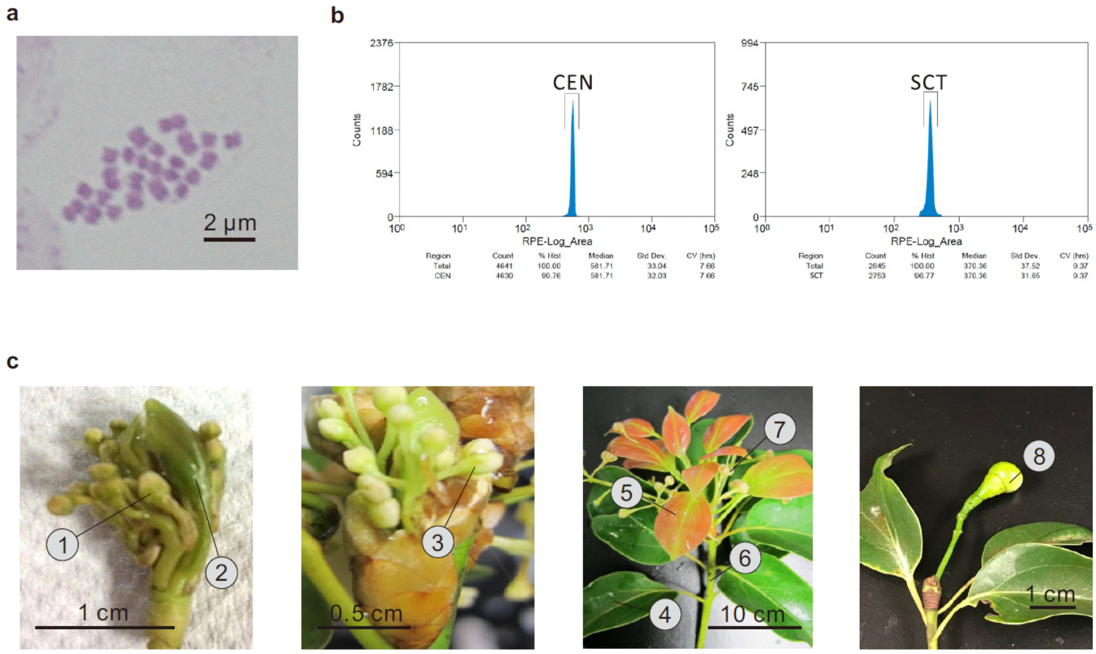

Supplementary Fig. 1. Chromosome biology and transcriptome sequencing of SCT. a, Basic fuchsin-stained root tip metaphase cells showing the chromosome number (2n = 24). Three independent staining and counts were carried out. **b,** flow cytometry estimation of *C. kanehirae* (SCT: 823.7±58.2 Mb/1C) genome size using chicken erythrocyte nuclei (CEN: 2.5 Gb) as the calibration standard. Two instruments, MoFlo XDP Cell Sorter (Beckman Coulter Life Science, Indianapolis, IN) and Attune NxT Flow Cytometer (Thermo Fisher Scientific Inc., Waltham, MA), were used to measure genome size using single leaf once and twice, respectively. The estimates by using the two instruments were similar and data obtained from the former is shown here. **c,** tissues used for RNA extraction with the stages of (1) flower buds enclosed within inflorescence bracts, (2) immature leaves enclosed within inflorescence bracts, (3) flower buds emerging from bracts, (4) old leaves, (5) young leaves in red, (6) stems, (7) opening flowers, and (8) fruits. Extractions were carried out once for every tissue.

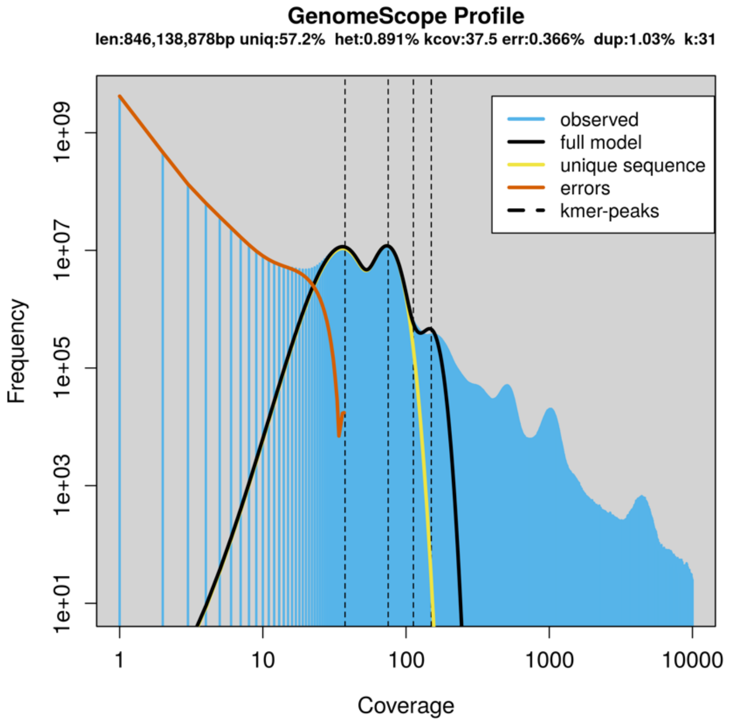

Supplementary Fig. 2. Estimate of genome size from Illumina paired end sequences of SCT using Genomescope[3].

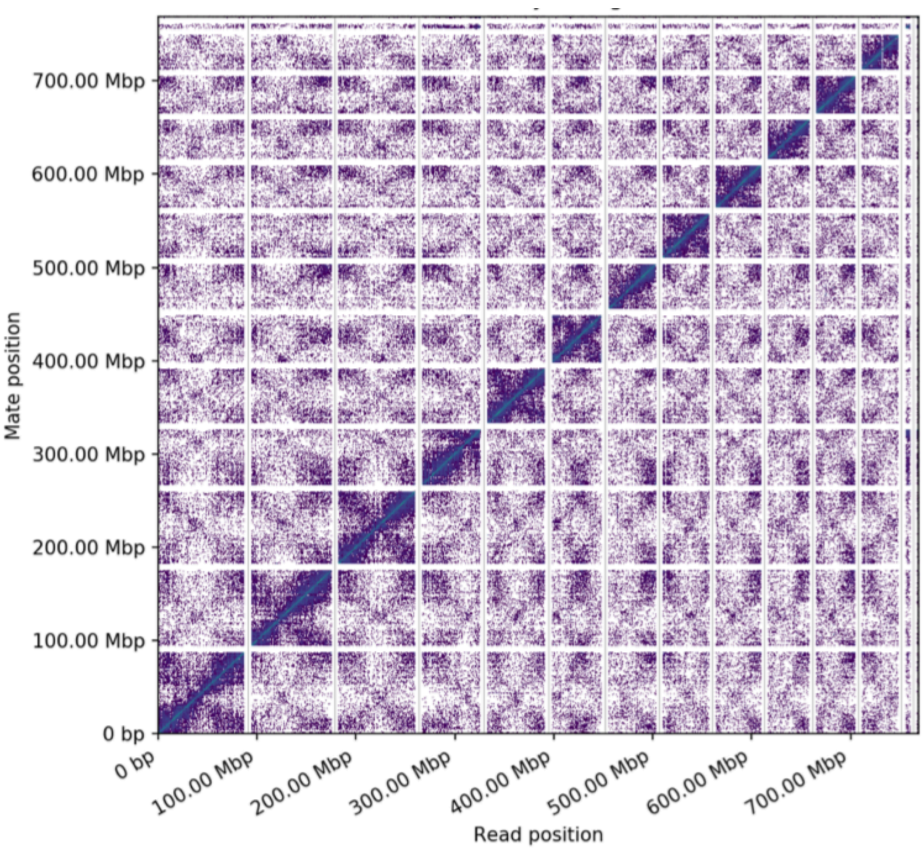

Supplementary Fig. 3. Contact matrices of the largest 12 scaffolds of the final SCT assembly. Hi-C reads were realigned back to the assembly and the mappings were converted to the dot intensity which indicate the likelihood of loci collocate in the nucleus.

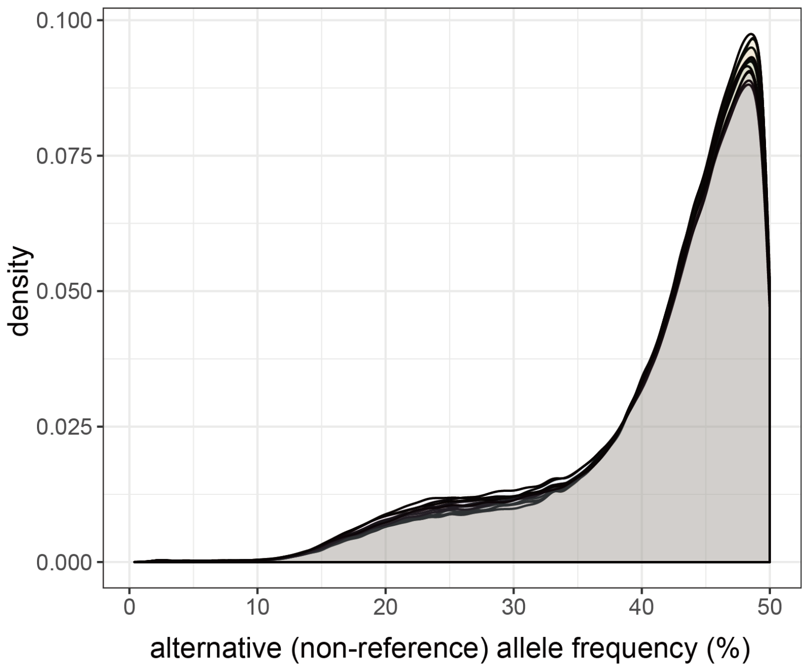

Supplementary Fig. 4. Distribution of alternative (non-reference) allele frequency on the largest 12 scaffolds of SCT. One density is shown for each scaffold.

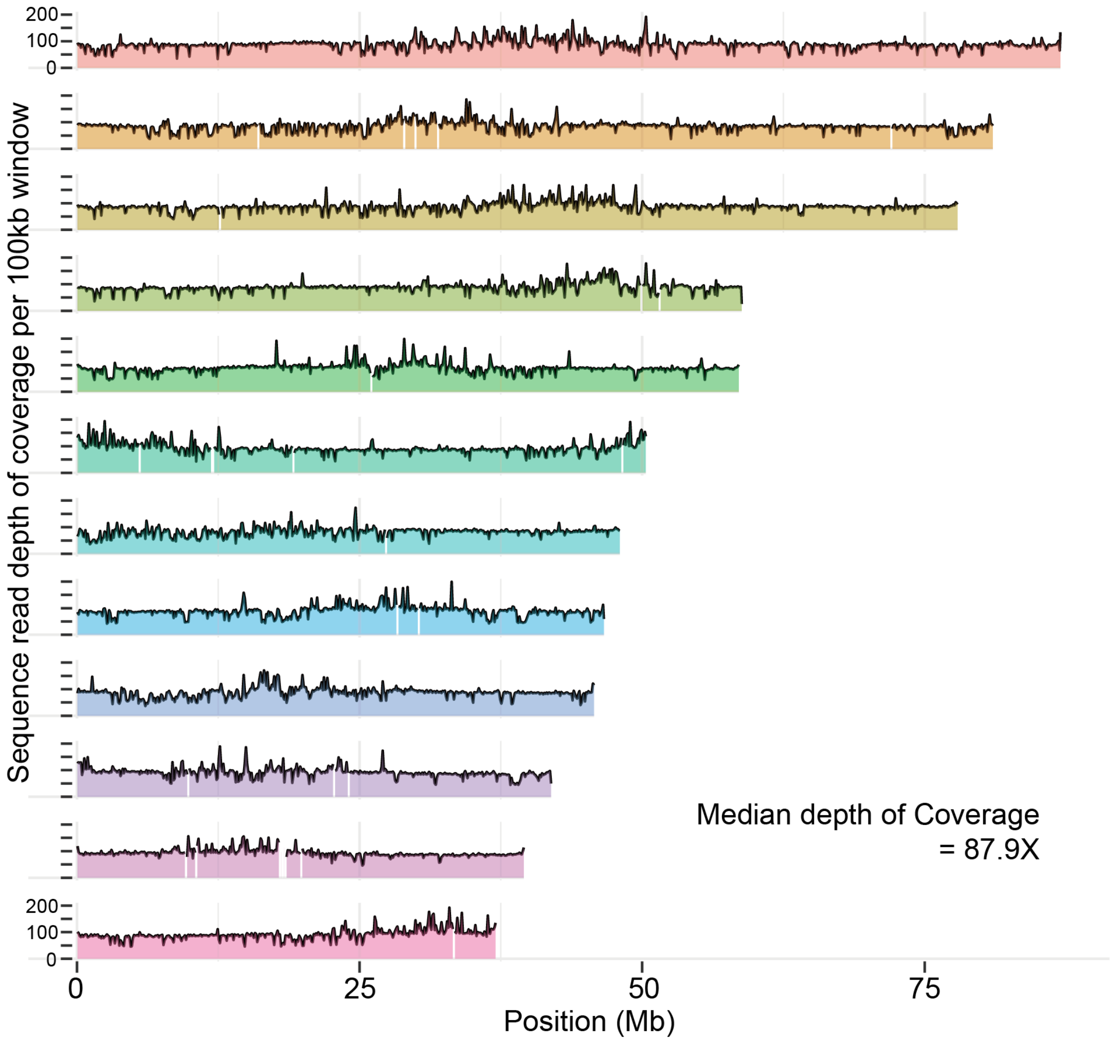

Supplementary Fig. 5. Sequence Mapping profile in SCT. Depth of Illumina genomic DNA sequencing coverage along the non-overlapping 100 kb windows of largest 12 scaffolds of SCT.

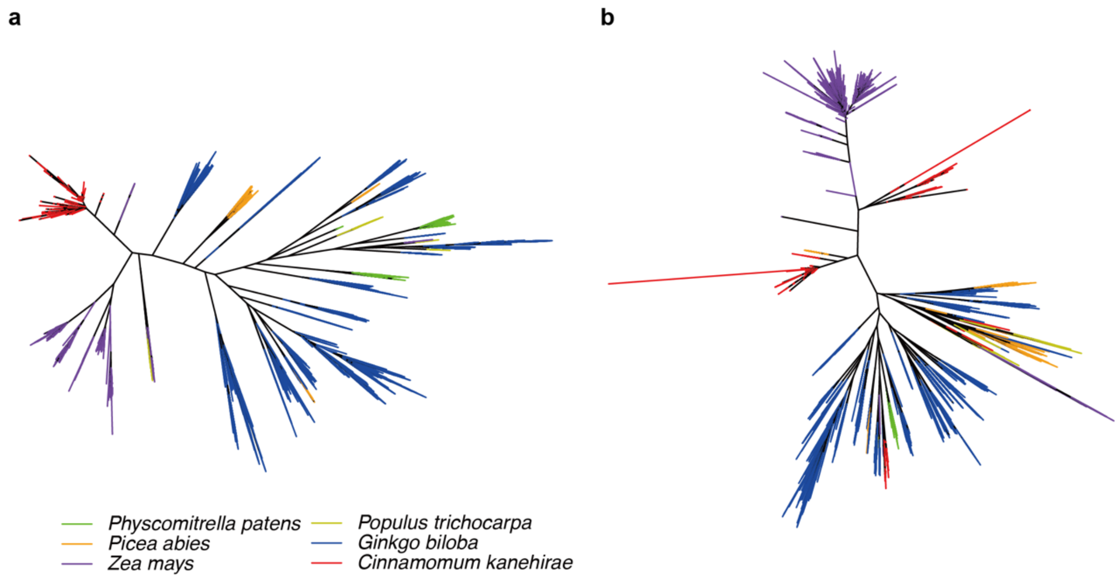

Supplementary Fig. 6. Phylogenetic relationships of LTR-RT domains. a, Inferred from Ty3/Gypsy. **b,** Inferred fromTy1/Copia LTR-RT domains. Branches are color-coded according to species.

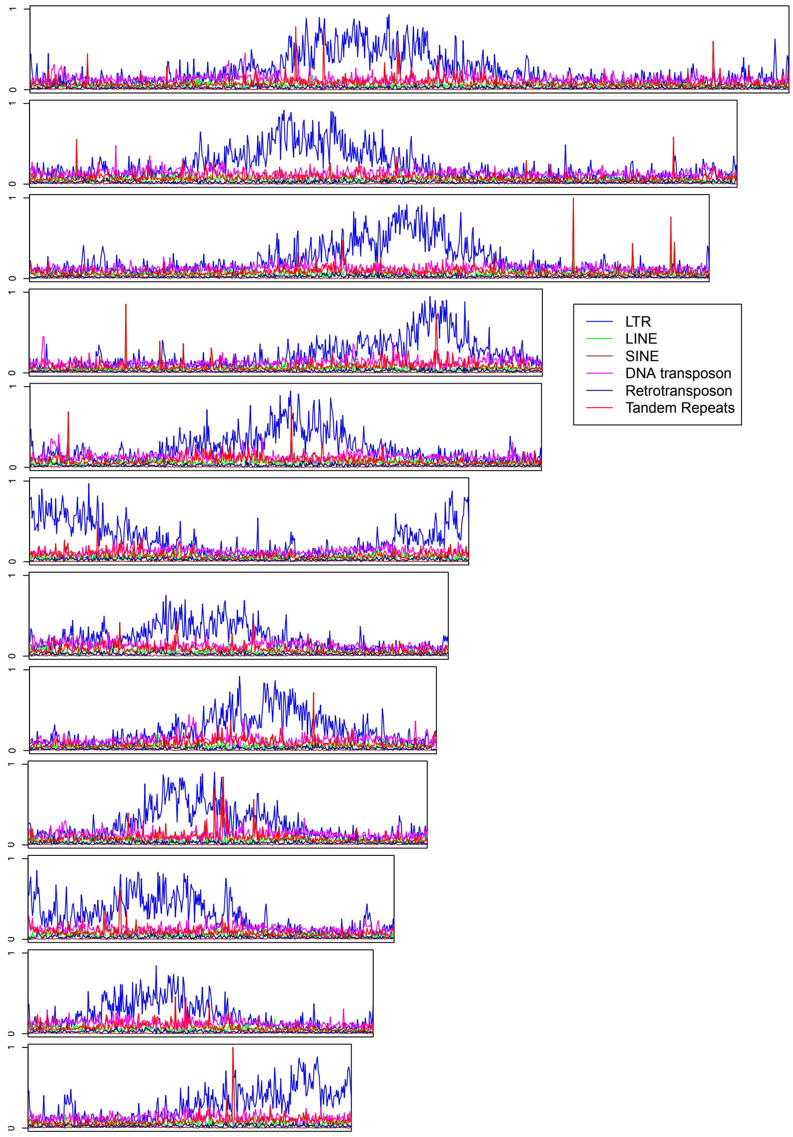

Supplementary Fig. 7. Distribution of TEs and genes along the 12 largest scaffolds of SCT.

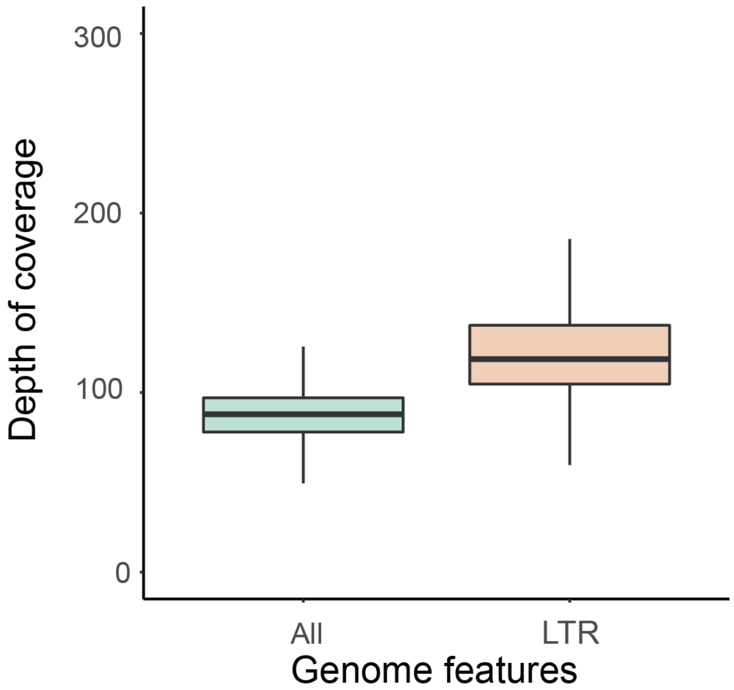

Supplementary Fig. 8. Boxplot of depth of coverage in LTR enriched windows (n= 249, Minium = 32.7X , Maxium = 236.1X, Median = 118.6X, 1st Quartile = 104.8X, 3rd Quartile = 137.4X,)versus all windows (n= 9,013, Min. = 0.08X , Max. = 15,042.3X, Median = 87.9X, 1st Qu. = 78.0X, 3rd Qu. = 97.0X)in the genome. The LTR enriched genome windows have a median of 118.6X, which is 35% higher.

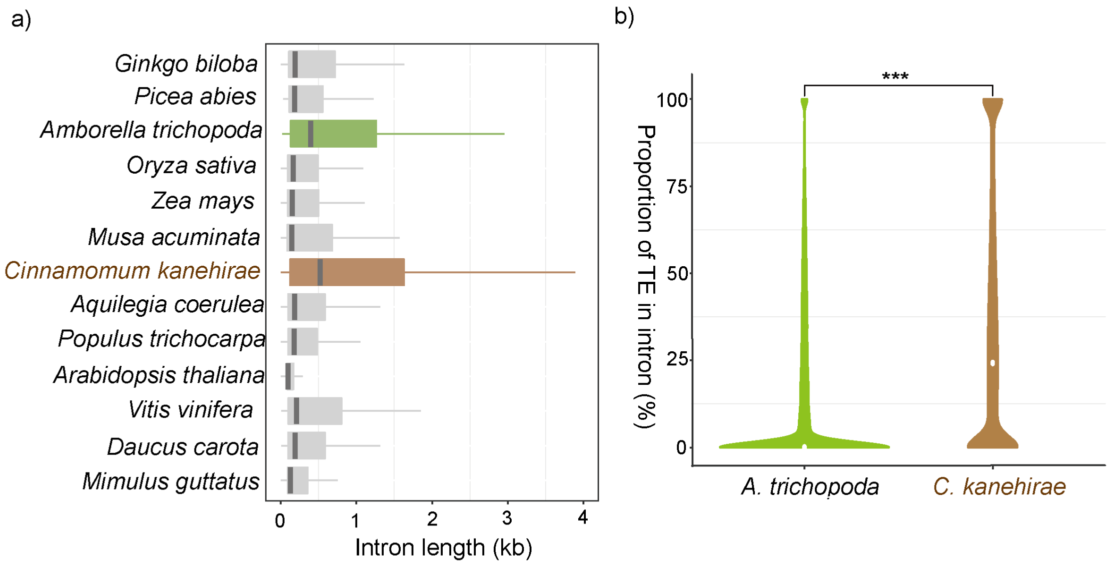

Supplementary Fig. 9. Intron dynamics of SCT. a, Distribution of intron length across plants (*Mimulus guttatus*, n = 117,749, Minimum = 3.0, 1st Quartile = 94.0, Median = 128.0, 3rd Quartile = 356.0, Maximum = 8135.0; *Daucus carota*, n = 128,674, Min. = 10.0, 1st Qu. = 97.0, Median = 193.0, 3rd Qu. = 584.0, Max. = 41367.0; *Vitis vinifera*, n = 135,706, Min. = 8.0, 1st Qu. = 102.0, Median = 211.0, 3rd Qu. = 802.0, Max. = 39915.0; *Arabidopsis thalian*, n = 118,640, Min. = 1.0, 1st Qu. = 85.0, Median = 99.0, 3rd Qu. = 167.0, Max. = 11601.0; *Populus tricocarpa*, n = 166,138, Min. = 1.0, 1st Qu. = 100.0, Median = 178.0, 3rd Qu. = 480.0, Max. = 10052.0; *Aquilegia coerulea*, n = 121,035, Min. = 1.0, 1st Qu. = 99.0, Median = 181.0, 3rd Qu. = 584.0, Max. = 10990.0; *Cinnamomum kanehirae*, n = 122,991, Min. = 2, 1st Qu. = 122, Median = 524, 3rd Qu. = 1629, Max. = 239861; *Musa acuminata*, n = 163,062, Min. = 1.0, 1st Qu. = 88.0, Median = 148.0, 3rd Qu. = 680.0, Max. = 25265.0; *Zea mays*, n = 167,171, Min. = 1.0, 1st Qu. = 93.0, Median = 155.0, 3rd Qu. = 500.0, Max. = 169079.0; *Oryza sativa*, n = 145,228, Min. = 4.0, 1st Qu. = 94.0, Median = 163.0, 3rd Qu. = 491.0, Max. = 18326.0; *Amborella trichopoda*, n = 82,937, Min. = 20, 1st Qu. = 134, Median = 394, 3rd Qu. = 1263, Max. = 175747; *Picea abies*, n = 107,313, Min. = 33.0, 1st Qu. = 112.0, Median = 182.0, 3rd Qu. = 558.0, Max. = 68268.0; *Ginkgo biloba*, n = 135,813, Min. = 1, 1st Qu. = 109, Median = 190, 3rd Qu. = 719, Max. = 1272917). **b,** Wilcoxon-rank sum test shows that the distribution of TE proportion in intron is significantly different (P = 4.79e-181; two-sided Wilcoxon rank sum test) between *C. kanehirae* and *A. trichopoda*. (*C. kanehirae*, n = 122,991, Min. = 0.00, 1st Qu. = 0.00, Median = 24.22, 3rd Qu. = 58.44, Max. = 100; *A. trichopoda*, n = 82,937, Min. = 0.00, 1st Qu. = 0.00, Median = 0.00, 3rd Qu. = 22.73, Max. = 100*)*

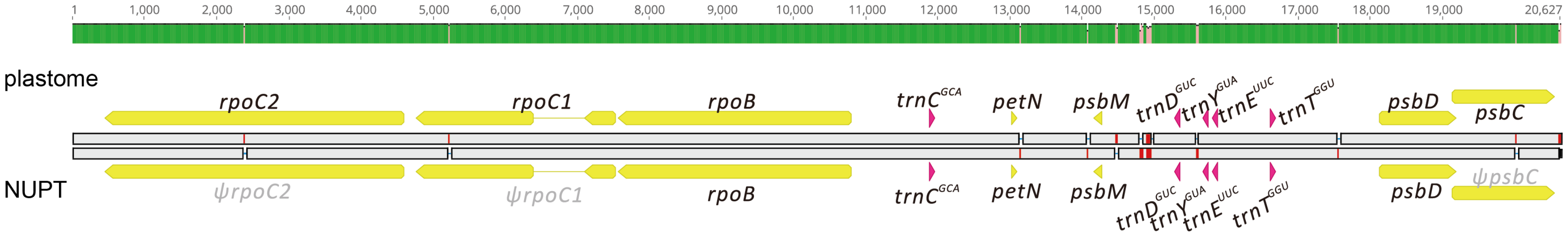

Supplementary Fig. 10. Sequence alignment of the longest NUPT found in SCT genome to its counterpart in plastome. The histograms represent sequence similarity colored as green (100%) and light pink (< 100%). Protein-coding and tRNA genes were denoted as yellow and pink arrows, respectively. Red and blue lines in the alignment indicate nucleotide differences and gaps between the two sequences. Pseudogenes were marked with psi (Ψ) symbol and labeled in gray fonts. Three out of the seven protein-coding genes were pseudogenized.

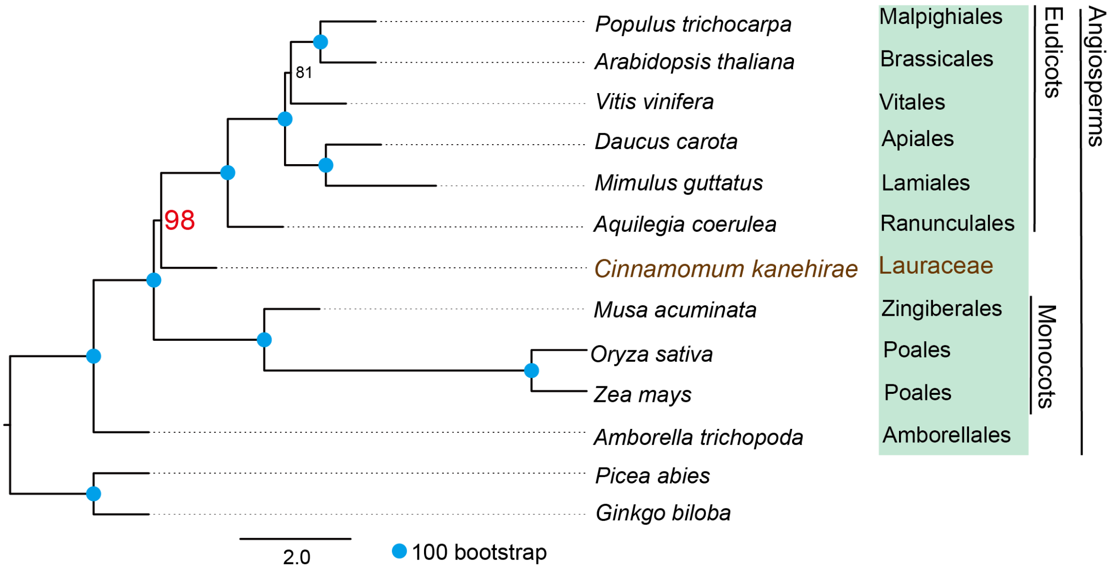

Supplementary Fig. 11. A species tree of 13 plant species based on the coalescence of gene trees constructed from protein sequence alignment of each of 211 single-copy orthologues using ASTRAL[4]. Number or blue dots on every node represent the proportion of gene trees that support each node.

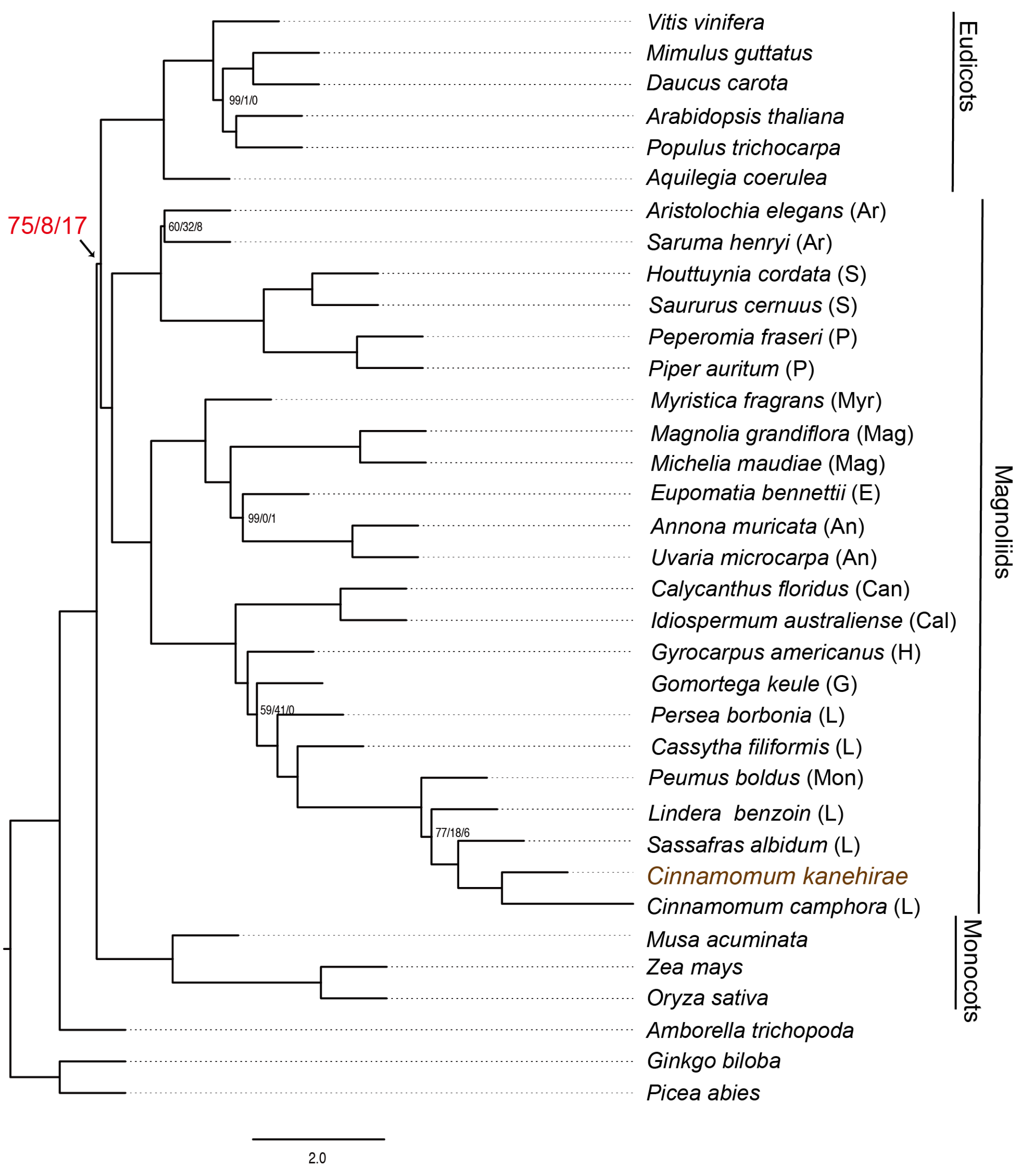

Supplementary Fig. 12. A species tree of 35 plant species based on the coalescence of gene trees using ASTRAL[4]. Gene trees were constructed from protein sequence alignment of each of 211 orthogroups inferred previously with the dataset of 13 species using RAxML[6] with 100 bootstrap replicates (options: -m PROTGAMMAILGF -f a). In each orthogroup, missing data were tolerated or one gene chosen from random for each of the additional species from 1KP[5]. Number on every node represent the local posterior probabilities of main topology and two alternatives. All nodes have 100/0/0 local posterior support unless stated otherwise. Bracket next species' name denote different families: Ar, Aristolochiaceae; S, Saururaceae; P, Piperaceae; Mon, Monimiaceae; Myr, Myristicaceae; Mag, Magnoliaceae; E, Eupomatiaceae; An, Annonaceae; Can, Canellaceae; Cal, Calycanthaceae; H, Hernandiaceae; G, Gomortegaceae; L, Lauraceae.

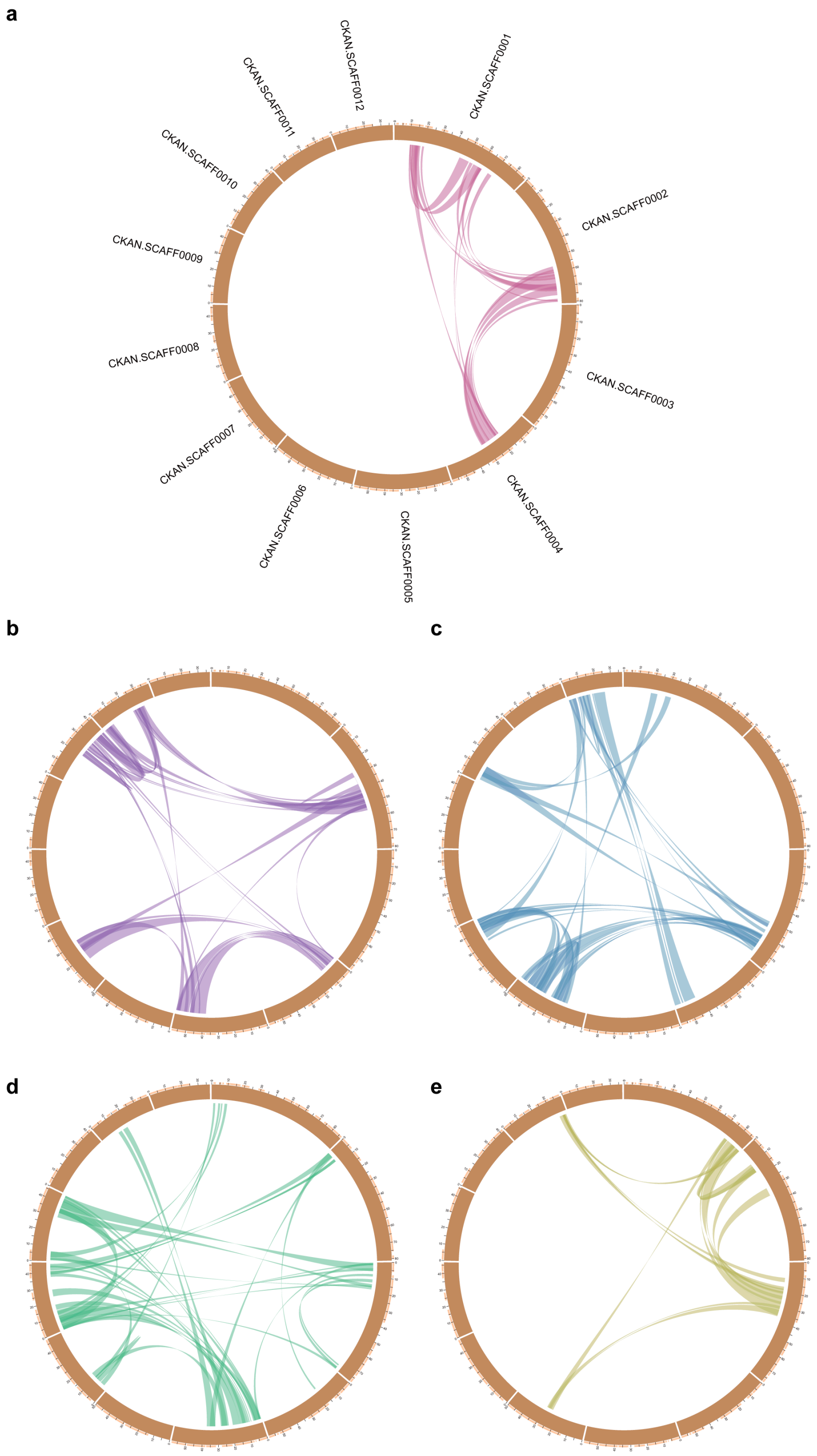

Supplementary Fig. 13. Assignment of synteny blocks into five linkage clusters.

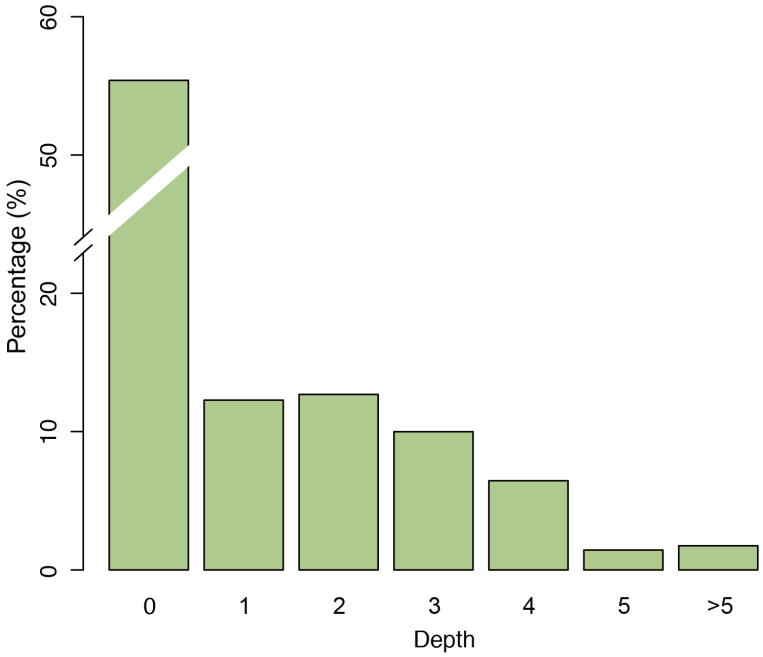

Supplementary Fig. 14. Observed depth of syntenic block coverage in the genome of SCT for every syntenic region of *A. trichopoda*. For example, 6.5% of *A. trichopoda* genome can be found in syntenic in four regions of SCT.

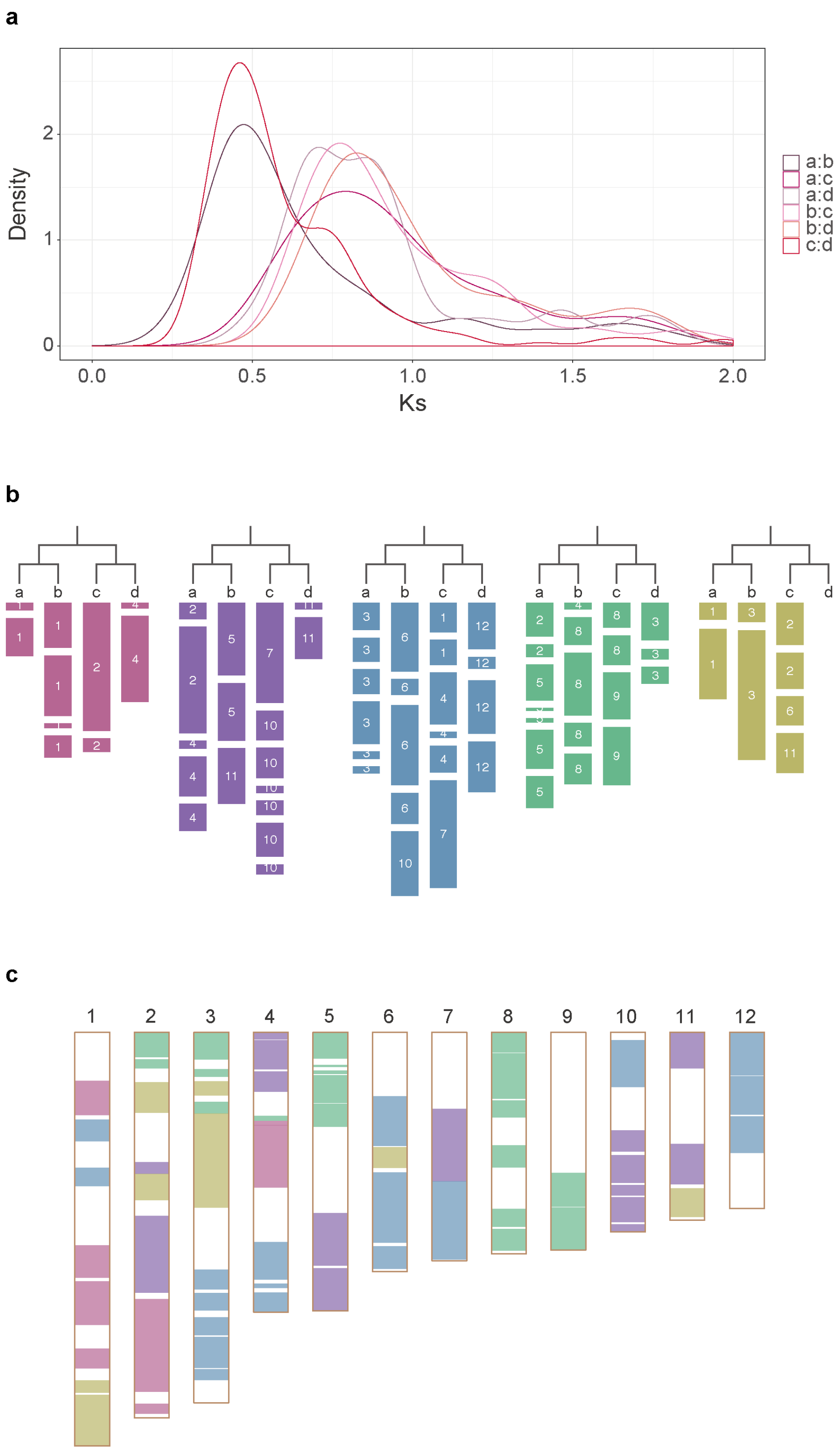

Supplementary Fig. 15. Intragenomic synteny block assignment and proposed karyotype evolution in SCT. **a,** Chromosomes in pairs that arose after the each WGD events were identified based on whether Ks distribution was peaked at ~0.46 (second WGD) or ~0.76 (first WGD). **b,** Proposed karyotype type of the synteny blocks **c.** Different color representing one of the five ancestral chromosomes plotted on the twelve SCT chromosomes.

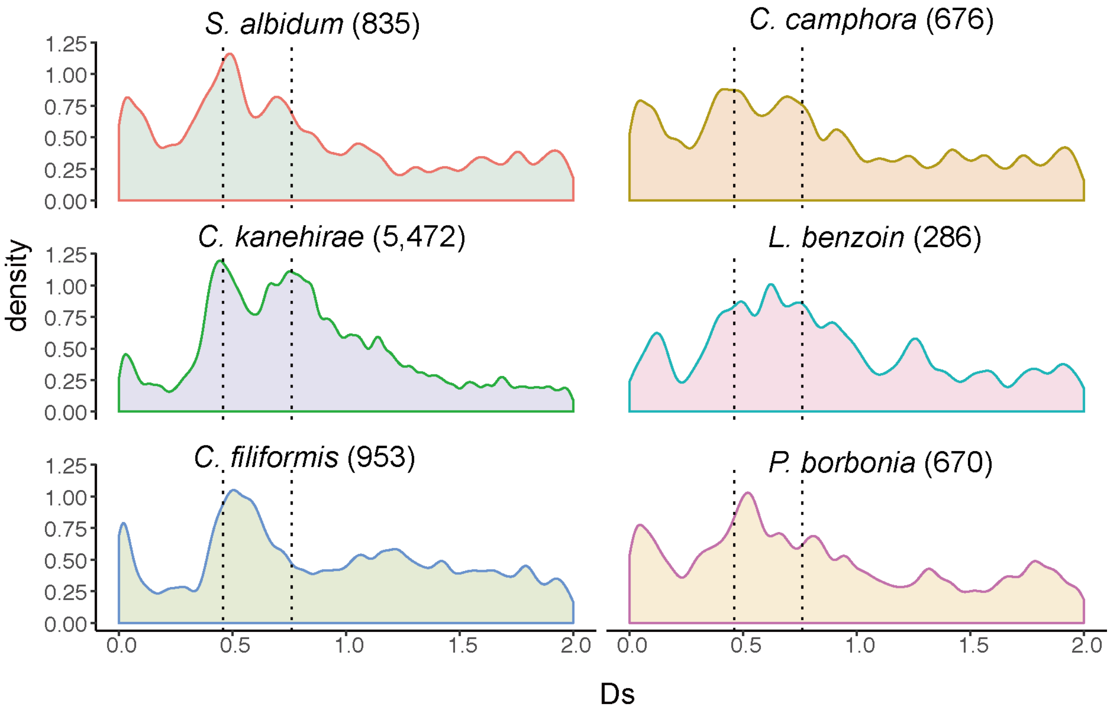

Supplementary Fig. 16. Density plots of synonymous substitutions (Ks) of Lauraceae in the 1KP[5] and SCT. Dashed lines denote the two Ks peaks observed in SCT. Number in brackets denote number of available pairwise intragenomic orthologues in each species.

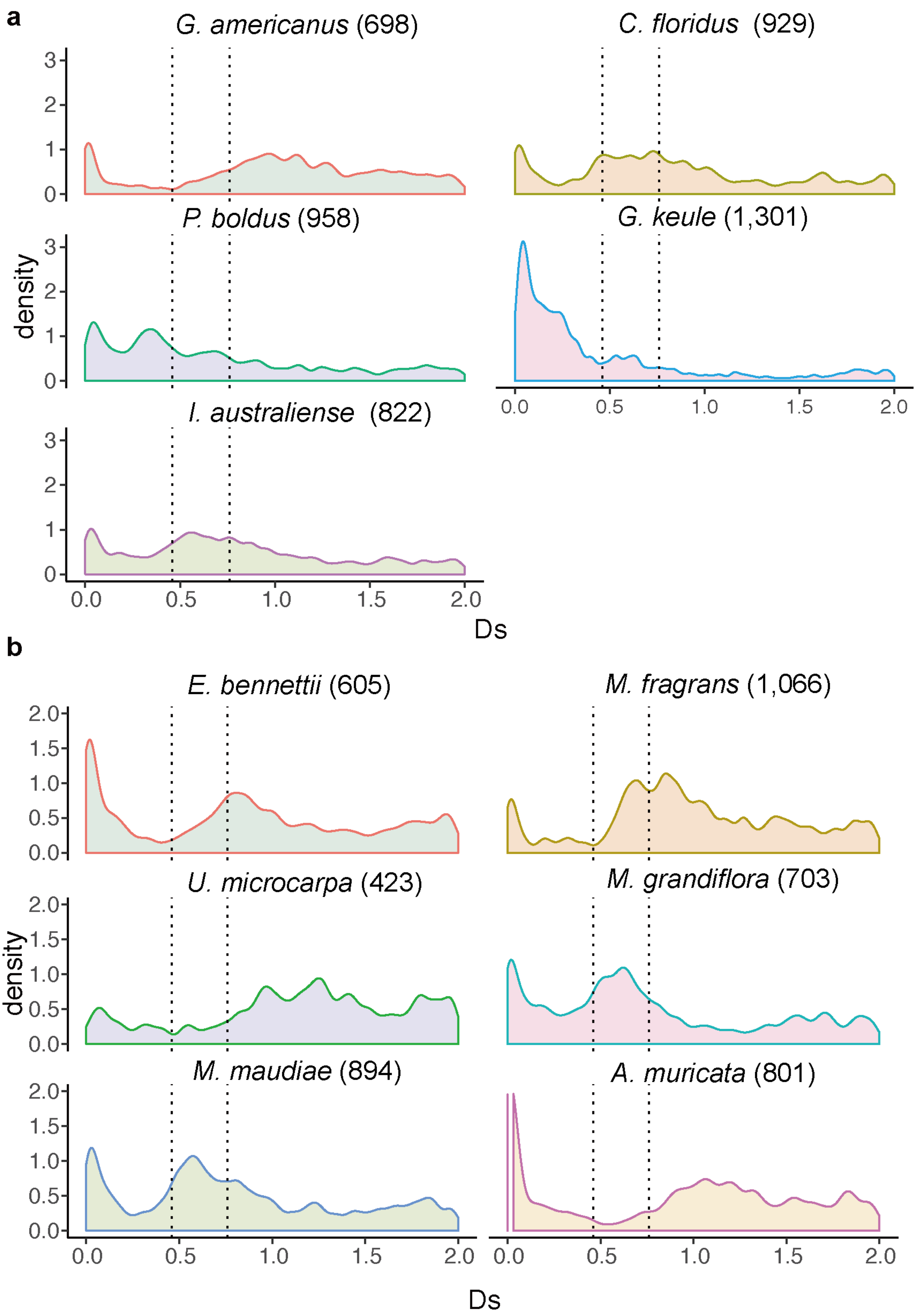

Supplementary Fig. 17. Density plots of synonymous substitutions (Ks) of intragenomic pairwise duplicates of a, Laurales outside Lauraceae. **b,** Magnoliales in the 1KP[5]. Dashed lines denote the two Ks peaks observed in SCT. Number in brackets denote number of available pairwise intragenomic orthologues in each species.

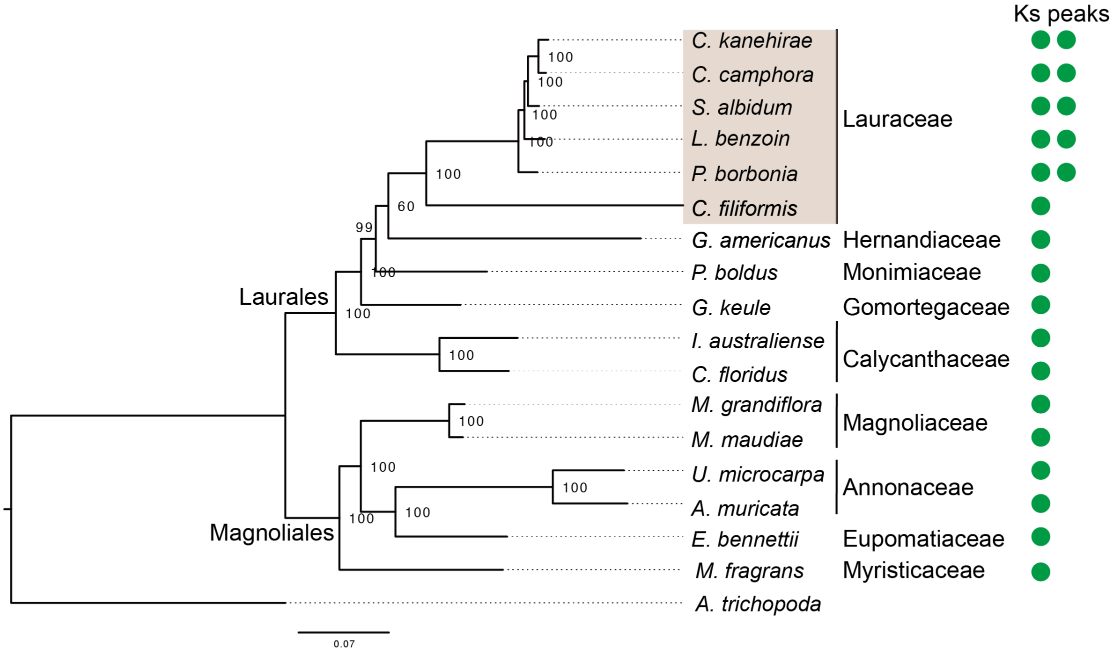

Supplementary Fig. 18. Phylogenomic analysis of Lauraceae WGD events. The two identified WGD events are placed on the phylogeny as circles. The tree shows the relationship of *C. kanehirae,* Laurales and Magnoliales from 1KP[5]. The maximum likelihood phylogeny was produced using concatenated amino acid alignment of 69 single copy orthologs using RAxML[6] with 500 bootstrap replicates (options: -m PROTGAMMAILGF -f a).

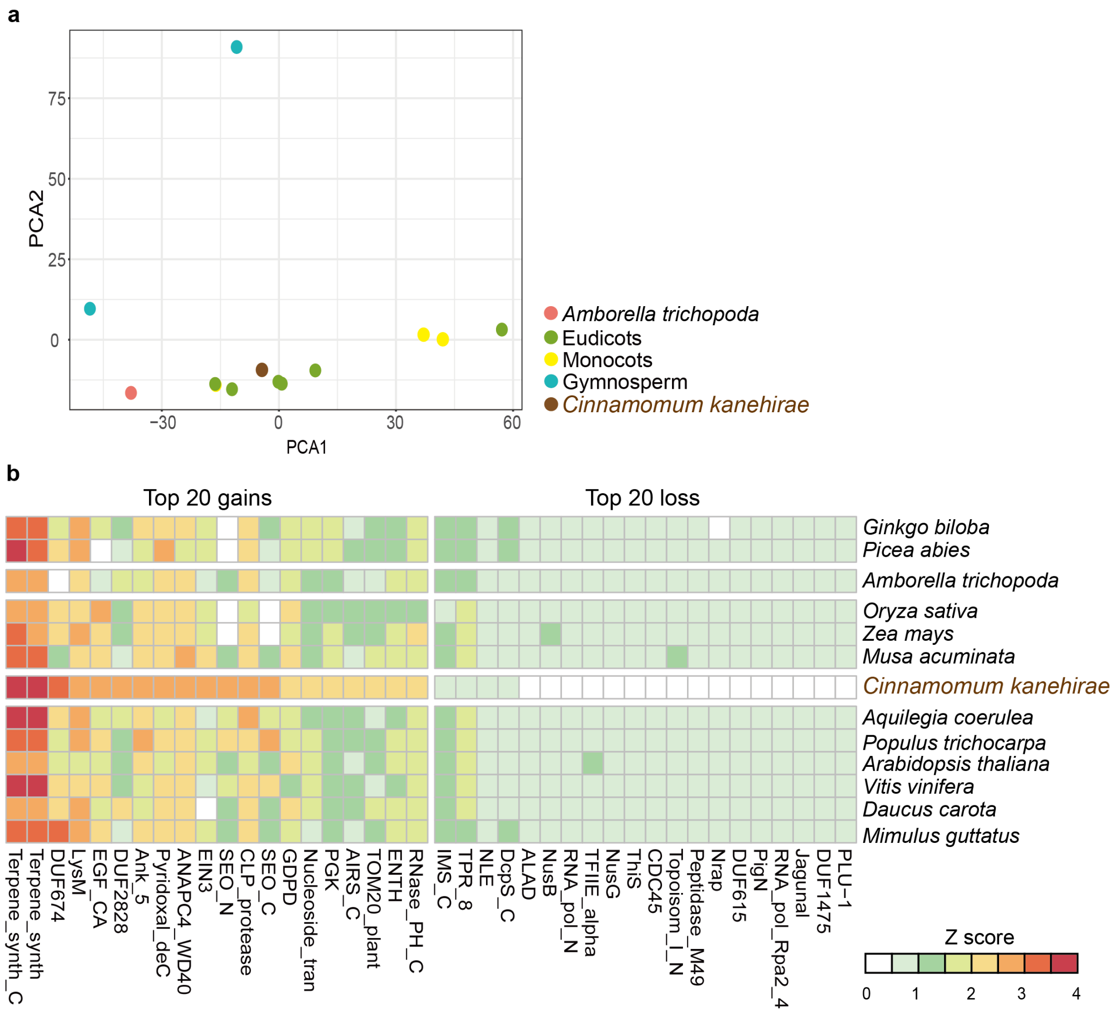

Supplementary Fig. 19. Protein family domain (Pfam) analysis across 13 plant species. a, Principal component analysis of numbers in 4,455 Pfams. **b,** Top 20 enrichment of Pfam gains and loss in *C. kanehirae* sorted by domain counts. For every Pfam a z-score was calculated for the corresponding abundance in each species. Only z-scores greater than 1.96 and -1.96 were included and shown in Supplementary Table 9.

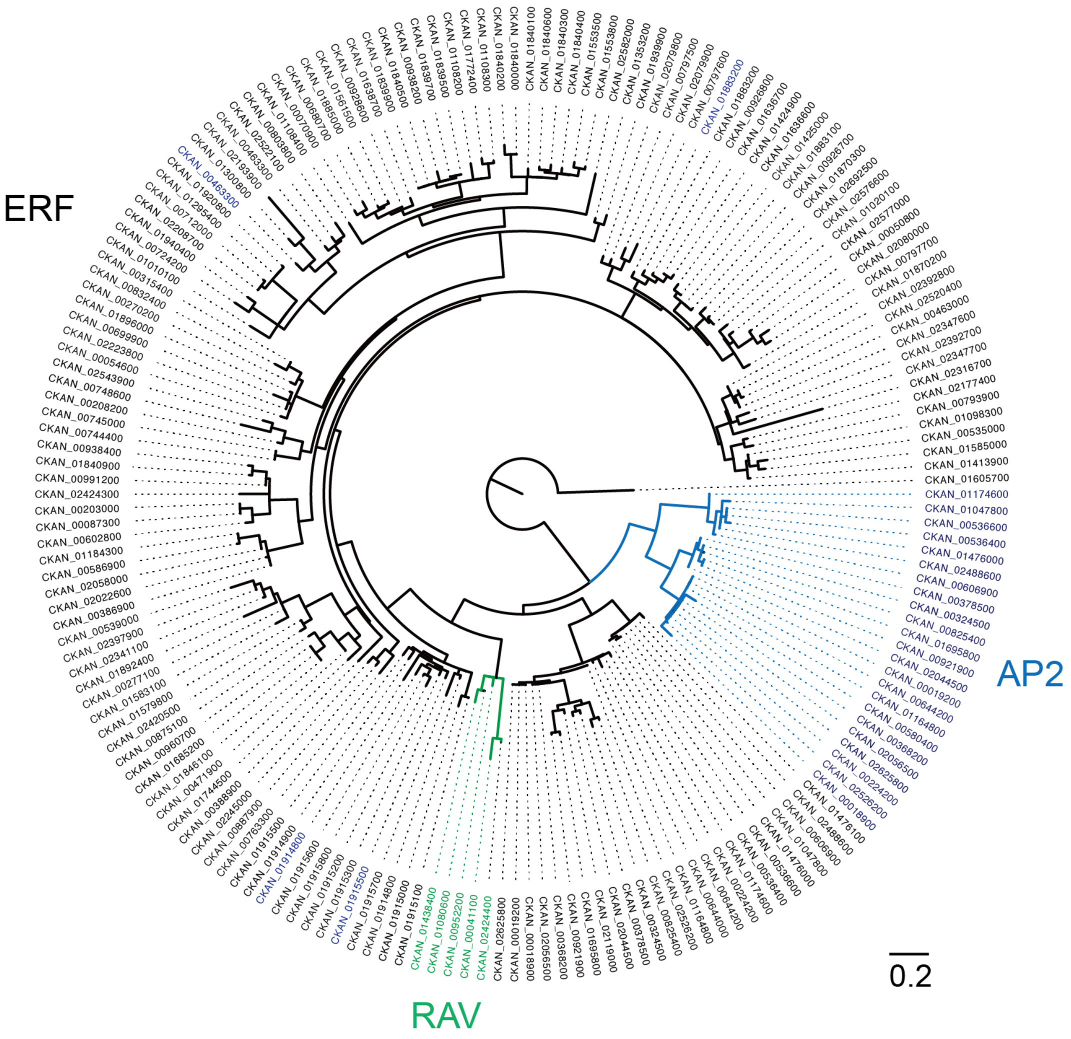

Supplementary Fig. 20. Phylogeny of APETALA2/Ethylene-responsive element binding protein (AP2/EREBP)-type transcription factors. The maximum likelihood phylogeny was constructed from alignment of the AP2 (PF00847) domain of proteins using RAxML[6]. The AP2/EREBP members were classified according to their phylogenetic positions and domain combination: AP2 with two AP2 domains (23 members); RAV (related to ABI3/VP) with a single AP2 and an additional B3 domain (four members), and rest being ethylene response factors (ERF; 150 members).

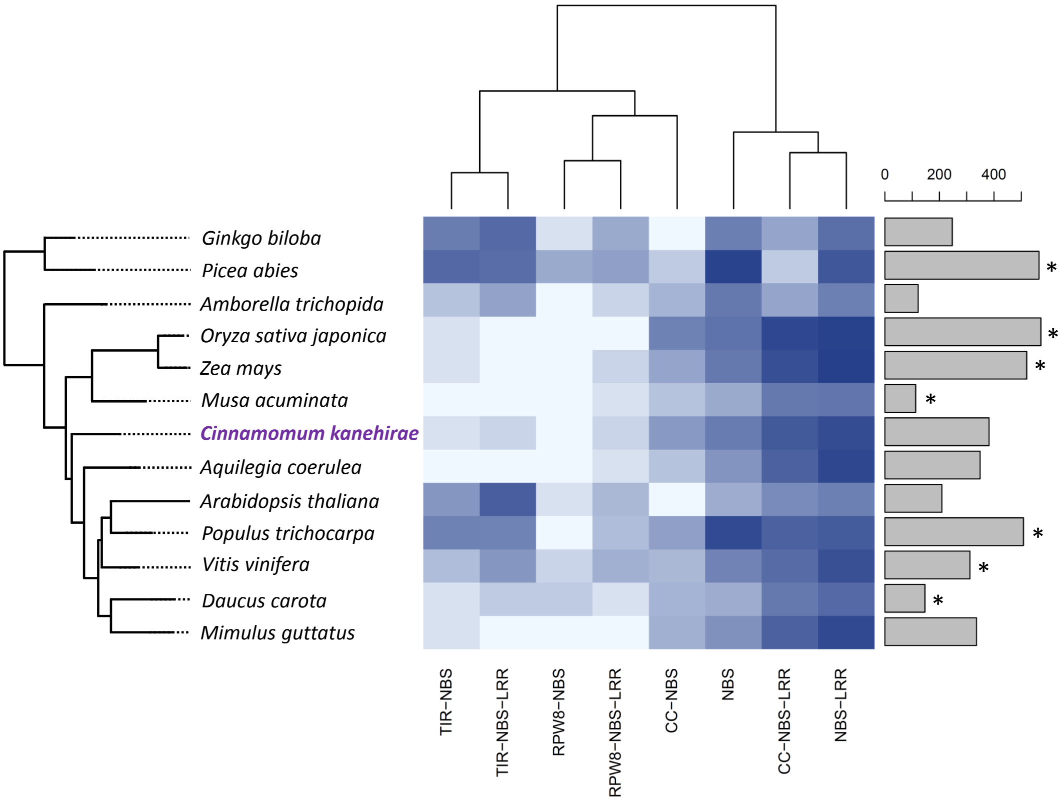

Supplementary Fig. 21. The distribution of resistance genes in the 13 species. The phylogenetic tree on the left side was derived from the tree built from 211 single copy genes. Darker and lighter colors in the heatmap indicate higher and lower numbers of corresponding resistance gene types, respectively. The hierarchical clustering tree at the top indicates the clustering of different resistance gene types across the species. The bar chart on the right side represents the total number of resistance genes for the 13 species. Asterisks (*) denote cultivated species.

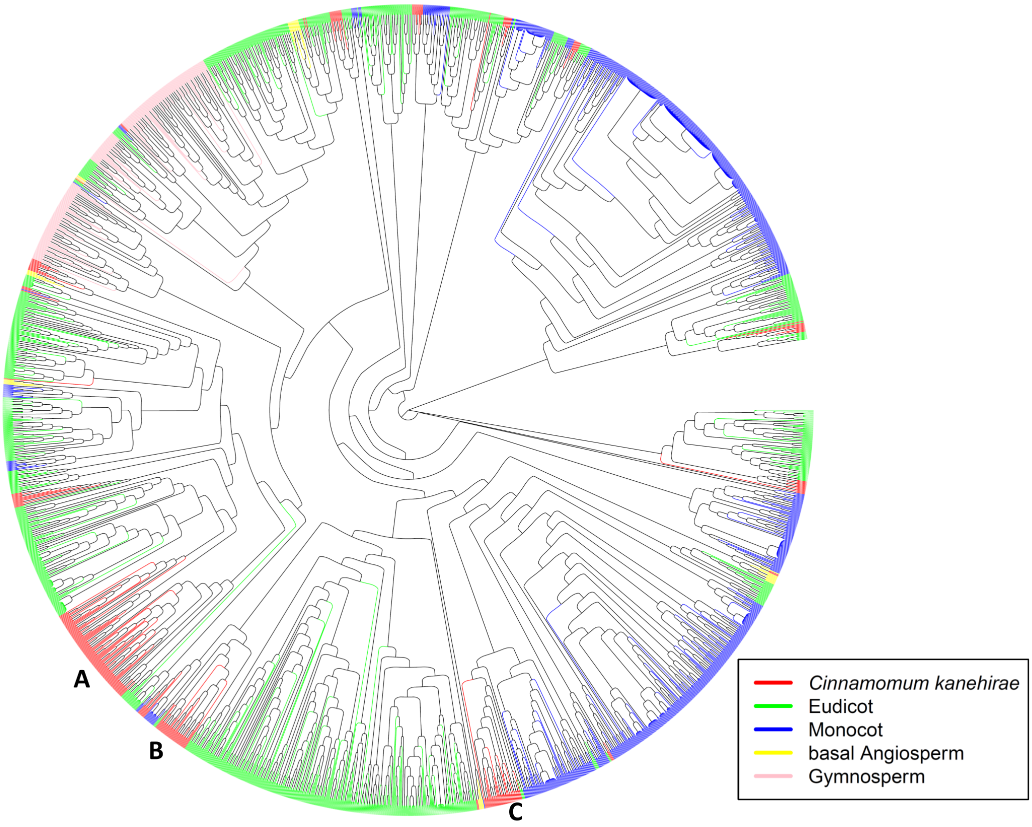

Supplementary Fig. 22. The phylogenetic tree of the NBS domain of the resistance genes. The three bold letters indicate major branching events of NBS domains in the resistance genes occurred in the evolutionary history of *C. kanehirae*.

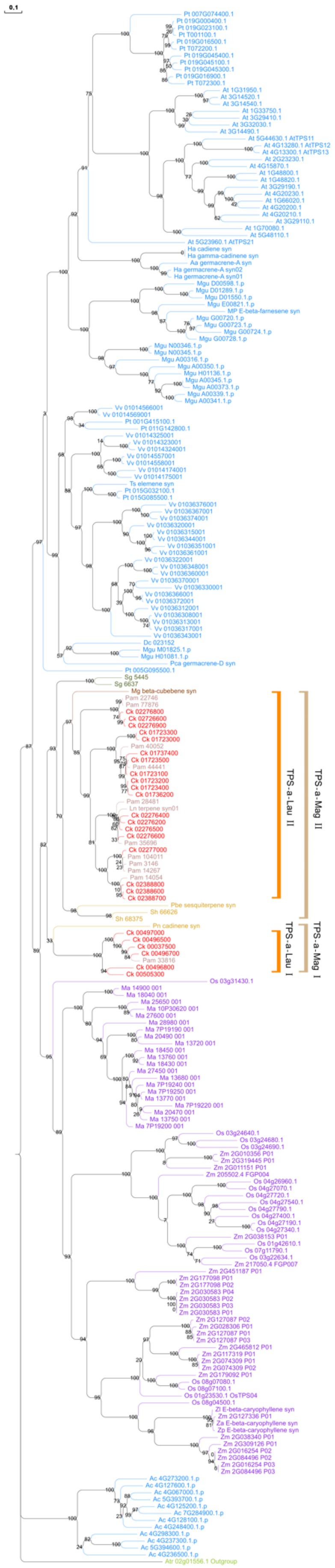
0.1
TPS-a-Lau II
TPS-a-Mag II
TPS-a-Lau I
TPS-a-Mag I

Supplementary Fig. 23. Phylogeny of the TPS-a subfamily from available magnoliids and the 13 sampled taxa. An *Amborella* TPS gene of TPS-g is chosen as the outgroup. The TPS-a subfamily of available magnoliids (including Piperales, Magnoliales, and Lauraceae) and Chloranthales (Sarcandra) formed a monophyletic clade with the TPS of monocots. Within the magnoliids, there are two well supported subclades —TPS-a-Mag I and TPS-a-Mag II—and one unresolved subclade containing only the cadinene synthase in *Piper nigrum*. The Lauraceae TPS genes form two monophyletic clades. The six members of TPS-a-Lau I are close to the cadinene synthase in *Piper nigrum*. The other 19 CkTPS-a formed four subgroups, each containing at least two TPS from *Persea* and one also containing TPS from *Laurus nobilis*. These four subclade are sister to the -cubene synthase in *Magnolia gradiflora* with 97% bootstraps support. The tree topology and placements of Lauraceous TPS data suggest that the TPS-a subfamily has duplicated at least five times in Lauraceae and 10 times within SCT. The 25 CkTPSs of TPS-a encode at least three kind of different TPSs. Aa, *Artemisia annua*; Ac, *Aquilegia coerulea*; Ag, *Abies grandis*; Am, *Antirrhinum majus*; Ar, *Agastache rugosa*; At, *Arabidopsis thaliana*; Atr, *Amborella trichopoda*; Cc, *C. camphora*; Ck, *C. kanehirae*; Cl, *Citrus limon*; Ct, *Cycas taitungensis*; Cte *C. tenuipile*; Cm, *C. micranthum*; Co, *C. osmophleum*; Es, *Ephedra sinica*; Gb, *Ginkgo biloba*; Ha, *Helianthus annuus*; Lc, *Litsea cubeba*; Ln, *Laurus nobilis*; Mc, *Magnolia champaca*; Mg, *M. grandiflora*; Mgu, *Mimulus guttatus*; Ml, *Mentha longifolia*; MP, *Mentha* x *Piperita*; Ms, *Mentha spicata*; Os, *Oryza sativa*; Pa, *Picea abies*; Pam, *Persea americana*; Pb, *Pinus banksiana*; Pbe, *Piper betle*; Pc, *Pogostemon cablin*; Pf, *Perilla frutescens*; Pg, *Picea glauca*; Pn, *Piper nigrum*; Sh, *Saruma henryi*; Sl, *Solanum lycopersicum*; So, *Salvia officinalis*; Sr, *Stevia rebaudiana*; Ss, *S. stenophylla*; Ts, *Toona sinensis*; Vv, *Vitis vinifera*; Za, *Zea mays*; Zl, *Z. luxurians*; Zp, *Z. perennis*.

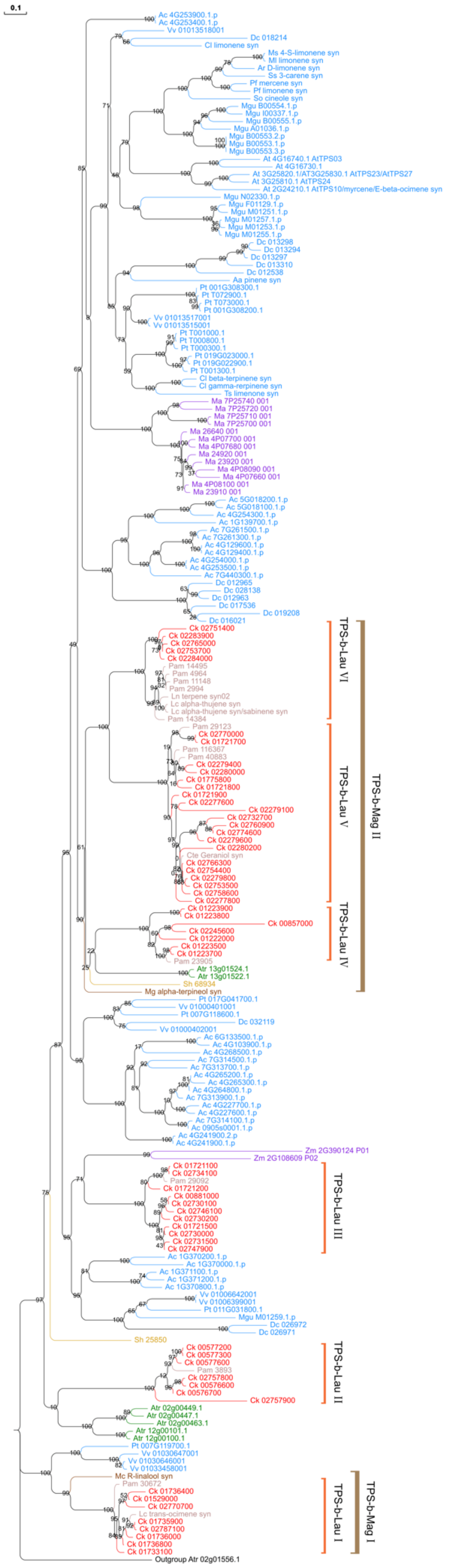
0.1
Ac 4G253900.1.p
Ac 4G253400.1.p
Vv 01013518001
Dc 018214
Cl limonene syn
Ms 4-S-limonene syn
Ml limonene syn
Ar D-limonene syn
Ss 3-carene syn
Pf mercene syn
Pf limonene syn
So cineole syn
Mgu B00554.1.p
Mgu I00337.1.p
Mgu B00555.1.p
Mgu A01036.1.p
Mgu B00553.2.p
Mgu B00553.1.p
Mgu B00553.3.p
At 4G16740.1 AtTPS03
At 4G16730.1
At 3G25820.1/AT3G25830.1 AtTPS23/AtTPS27
At 3G25810.1 AtTPS24
At 2G24210.1 AtTPS10/myrcene/E-beta-ocimene syn
Mgu N02330.1.p
Mgu F01129.1.p
Mgu M01251.1.p
Mgu M01257.1.p
Mgu M01253.1.p
Mgu M01255.1.p
Dc 013298
Dc 013294
Dc 013297
Dc 013310
Dc 012538
Aa pinene syn
Pt 001G308300.1
Pt T072900.1
Pt T073000.1
Pt 001G308200.1
Vv 01013517001
Vv 01013515001
Pt T001000.1
Pt T000800.1
Pt T000300.1
Pt 019G023000.1
Pt 019G022900.1
Pt T001300.1
Cl beta-terpinene syn
Cl gamma-rerpinene syn
Ts limenone syn
Ma 7P25740 001
Ma 7P25720 001
Ma 7P25710 001
Ma 7P25700 001
Ma 26640 001
Ma 4P07700 001
Ma 4P07680 001
Ma 24920 001
Ma 23920 001
Ma 4P08090 001
Ma 4P07660 001
Ma 4P08100 001
Ma 23910 001
Ac 5G018200.1.p
Ac 5G018100.1.p
Ac 4G254300.1.p
Ac 1G139700.1.p
Ac 7G261500.1.p
Ac 7G261300.1.p
Ac 4G129600.1.p
Ac 4G129400.1.p
Ac 4G254000.1.p
Ac 4G253500.1.p
Ac 7G440300.1.p
Dc 012965
Dc 028138
Dc 012963
Dc 017536
Dc 019208
Dc 016021
Ck 02751400
Ck 02283900
Ck 02765000
Ck 02753700
Ck 02284000
Pam 14495
Pam 4964
Pam 11148
Pam 2994
Ln terpene syn02
Lc alpha-thujene syn
Lc alpha-thujene syn/sabinene syn
Pam 14384
Pam 29123
Ck 02770000
Ck 01721700
Pam 116367
Pam 40883
Ck 02279400
Ck 02280000
Ck 01775800
Ck 01721800
Ck 01721900
Ck 02277600
Ck 02279100
Ck 02732700
Ck 02760900
Ck 02774600
Ck 02279600
Ck 02280200
Cte Geraniol syn
Ck 02766300
Ck 02754400
Ck 02279800
Ck 02753500
Ck 02758600
Ck 02277800
Ck 01223900
Ck 01223800
Ck 00857000
Ck 02245600
Ck 01222000
Ck 01223500
Ck 01223700
Pam 23905
Atr 13g01524.1
Atr 13g01522.1
Sh 68934
Mg alpha-terpineol syn
Pt 017G041700.1
Vv 01000401001
Pt 007G118600.1
Dc 032119
Vv 01000402001
Ac 6G133500.1.p
Ac 4G103900.1.p
Ac 4G268500.1.p
Ac 7G314500.1.p
Ac 7G313700.1.p
Ac 4G265200.1.p
Ac 4G265300.1.p
Ac 4G264800.1.p
Ac 7G313900.1.p
Ac 4G227700.1.p
Ac 4G227600.1.p
Ac 7G314100.1.p
Ac 0905s0001.1.p
Ac 4G241900.2.p
Ac 4G241900.1.p
Zm 2G390124 P01
Zm 2G108609 P02
Ck 01721100
Ck 02734100
Pam 29092
Ck 01721200
Ck 00881000
Ck 02730100
Ck 02746100
Ck 02730200
Ck 01721500
Ck 02730000
Ck 02731500
Ck 02747900
Ac 1G370200.1.p
Ac 1G370000.1.p
Ac 1G371100.1.p
Ac 1G371200.1.p
Ac 1G370800.1.p
Vv 01006642001
Vv 01006399001
Pt 011G031800.1
Mgu M01259.1.p
Dc 026972
Dc 026971
Sh 25850
Ck 00577200
Ck 00577300
Ck 00577600
Pam 3893
Ck 02757800
Ck 00576600
Ck 00576700
Ck 02757900
Atr 02g00449.1
Atr 02g00447.1
Atr 02g00463.1
Atr 12g00101.1
Atr 12g00100.1
Pt 007G119700.1
Vv 01030647001
Vv 01030646001
Vv 01033458001
Mc R-linalool syn
Pam 30672
Ck 01736400
Ck 01529000
Ck 02770700
Lc trans-ocimene syn
Ck 01735900
Ck 02787100
Ck 01736000
Ck 01736800
Ck 01733100
Outgroup Atr 02g01556.1
TPS-b-Lau VI
TPS-b-Lau V
TPS-b-Lau IV
TPS-b-Mag II
TPS-b-Lau III
TPS-b-Lau II
TPS-b-Lau I
TPS-b-Mag I

Supplementary Fig. 24. Phylogeny of the TPS-b subfamily from available magnoliids and the 13 sampled taxa. An *Amborella* TPS-g sequence is chosen as the outgroup (the same as in the TPS-a). Two monophyletic magnollids-TPS-b are resolved. A total of 58 CkTPSs form at least six Lauraceae subgroups in TPS-b. In detail, TPS-b-Lau I was clustered with a R-linalool synthase from *Magnolia champaca*, which is shown as TPS-b-Mag I in the figure, and it clusters with the eudicot-specific subgroup with 100% bootstrap support. TPS-b-Lau II contains seven CkTPSs and one PaTPS, and five *Amborella* TPSs but with only 10% bootstrap values. TPS-b-Lau III, two maize TPSs, and a well-supported eudicot clade (containing eleven TPSs) together form a highly supported cluster (95% bootstrap replicates). TPS-b-Mag II is divided into three subgroups: TPS-b-Lau IV, TPS-b-Lau V, and TPS-b-Lau VI. This tree topology suggests that 32 paralogous duplication events have occurred in CkTPS genes.

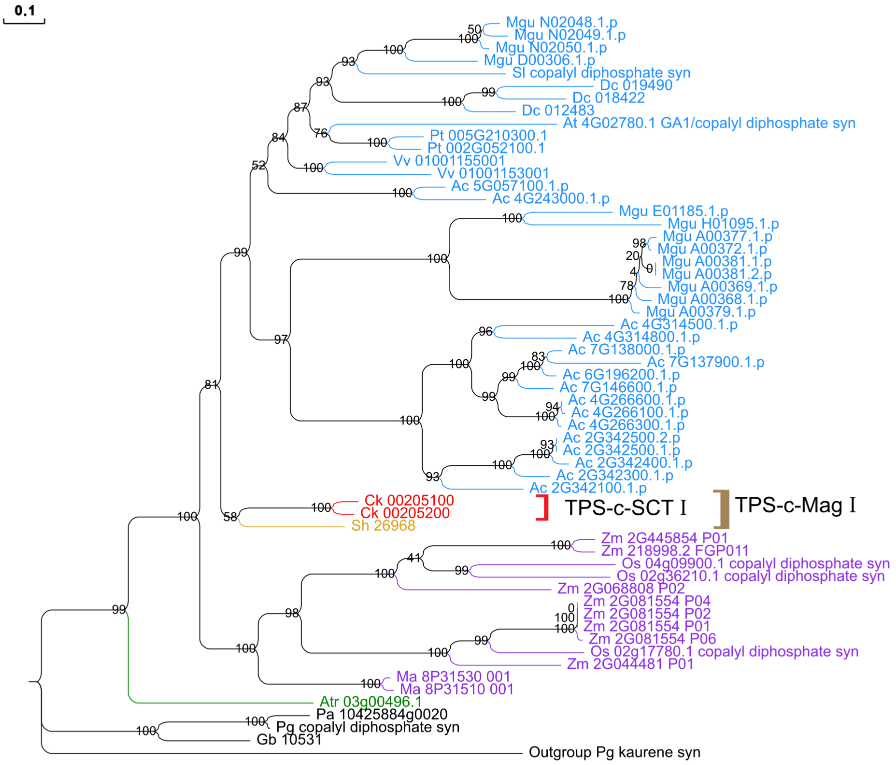

Supplementary Fig. 25. Phylogeny of the TPS-c subfamily from available magnoliids and the 13 sampled taxa. A kaurene synthase gene in *Picea glauca* from the TPS-e subfamily is chosen as the outgroup. Two CkTPSs and one *Saruma* TPS are clustered together in one clade, labeled "TPS-c-Mag I" in the figure. In subfamily TPS-c, the magnoliids-clade is clustered with eudicots. This tree topology suggests that there is only one paralogous duplication.

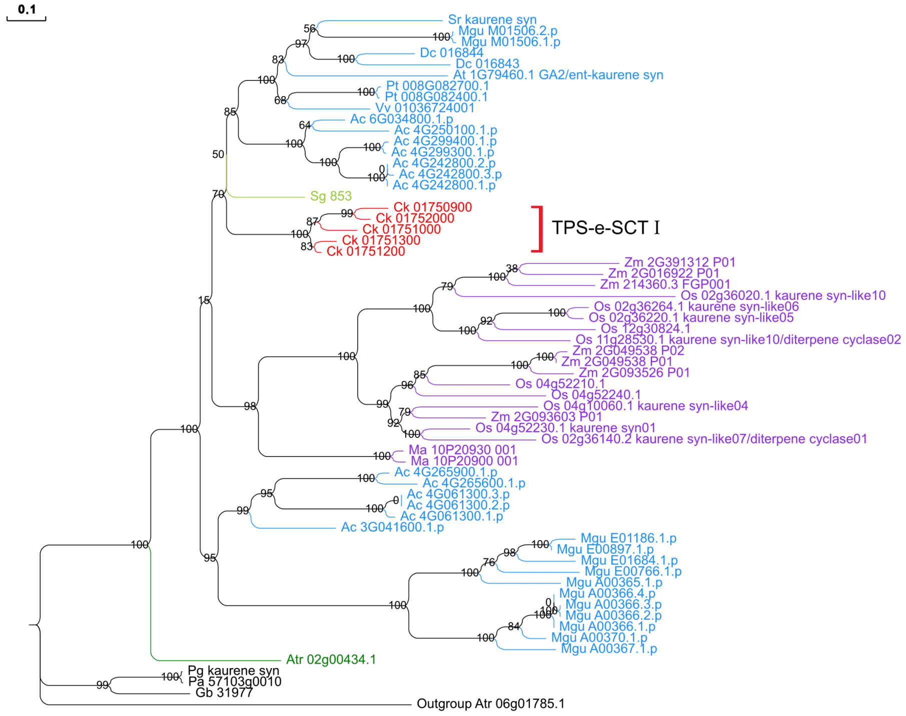

Supplementary Fig. 26. Phylogeny of the TPS-e subfamily from available magnoliids and the 13 sampled taxa. An *Amborella* TPS of TPS-f was chosen as the outgroup. All sampled eudicots form three monophyletic clades, and the TPSs of all three monocots form a monophyletic group. The five CkTPSs also form a monophyletic clade, TPS-e-SCT I, which is sister to one *Sarcandra* TPS and a group of eudicot-specific TPS but with low bootstrap supports (70%). The TPS-e-SCT I likely had four paralogous duplications based on this tree topology.

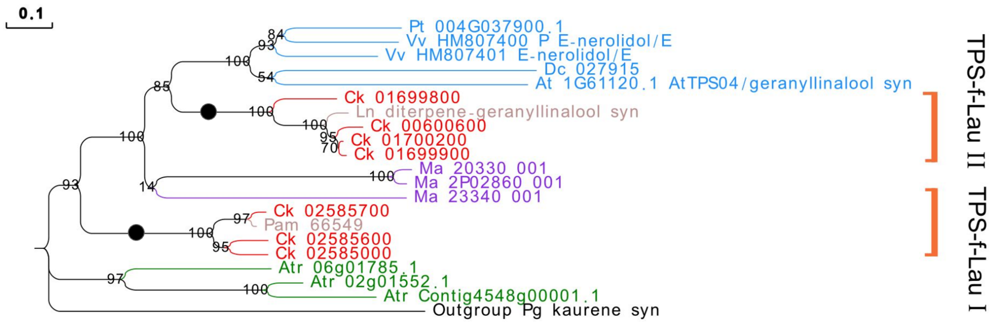

Supplementary Fig. 27. Phylogenic analysis of the TPS-f subfamily from all available magnoliids and the 13 sampled taxa. As TPS-f subfamily genes were not found in gymnosperms, a kaurene synthase gene in *Picea glauca* from the subfamily TPS-e was chosen as the outgroup. Two Lauraceae-specific groups, TPSf-Lau I and TPS-f-Lau II, are resolved. TPS-f-Lau I is sister to a large clade containing three banana TPSs, TPS-f-Lau II, and a eudicots clade consisting of five TPSs, and the latter two form a well-supported monophyletic group (85%). The four CkTPS genes in TPS-f-Lau II likely code for the geranyl linalool synthase as they are clustered with the geranyl linalool synthase gene of *Laurus nobilis*. This tree topology suggests that there are a total of three CkTPS-specific duplication events. The branches labeled with circles were used to detect positive selection under the branch-site model analysis (Supplementary Table 13).

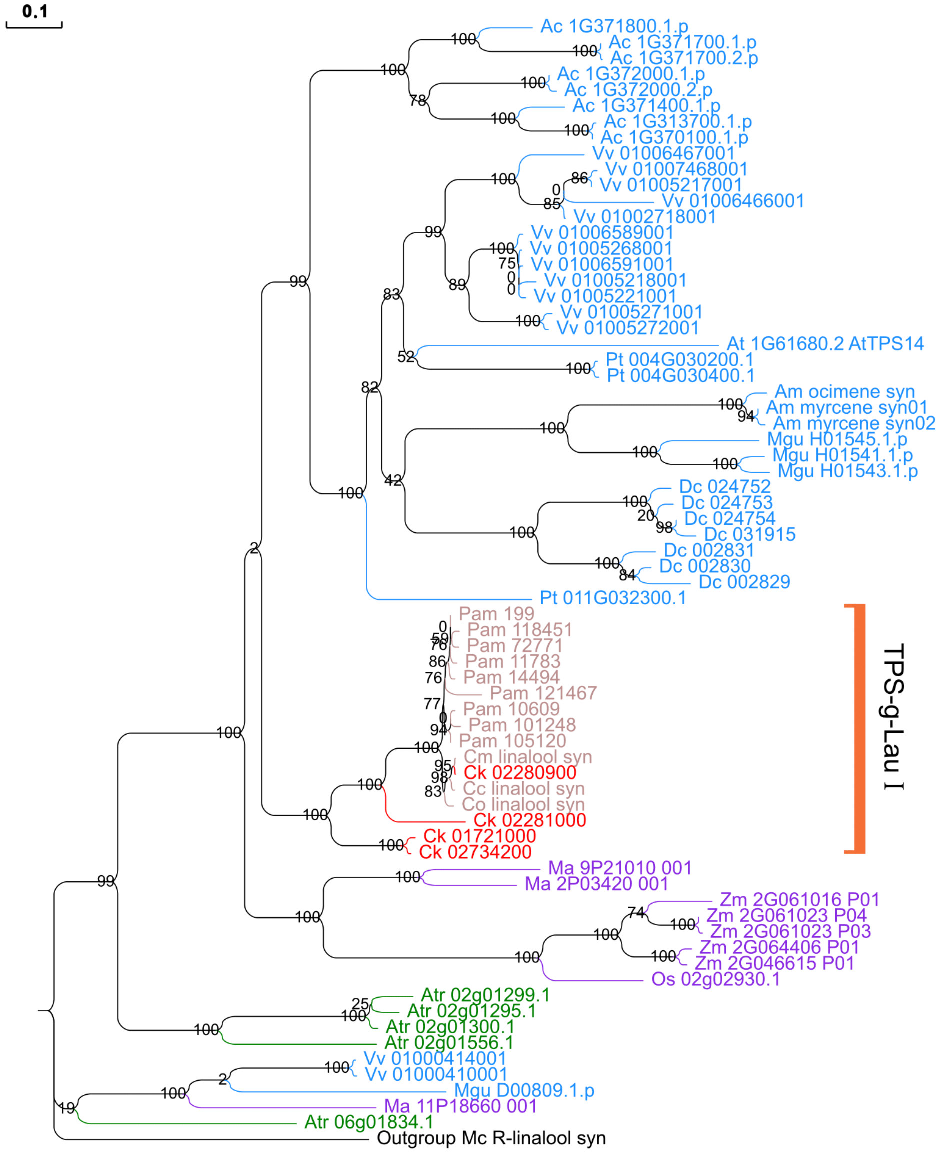

Supplementary Fig. 28. Phylogeny of the TPS-g subfamily from all available magnoliids and the 13 sampled taxa. As gymnosperms do not have TPS-g subfamily genes, a R-linalool synthase in *Magnolia champaca* from TPS-b was chosen as the outgroup. The TPS-g-Lau I is resolved as a monophyletic clade and subdivided into three subclades, one *PaTPs*-specific group, one *Cinnamomum*-specific group (all linalool synthase), and two CkTPS groups. However, the relationships among TPS-g-Lau I, monocots, and eudicots are not resolved. This tree topology suggests that there is only one CkTPS-specific duplication (paralogous).

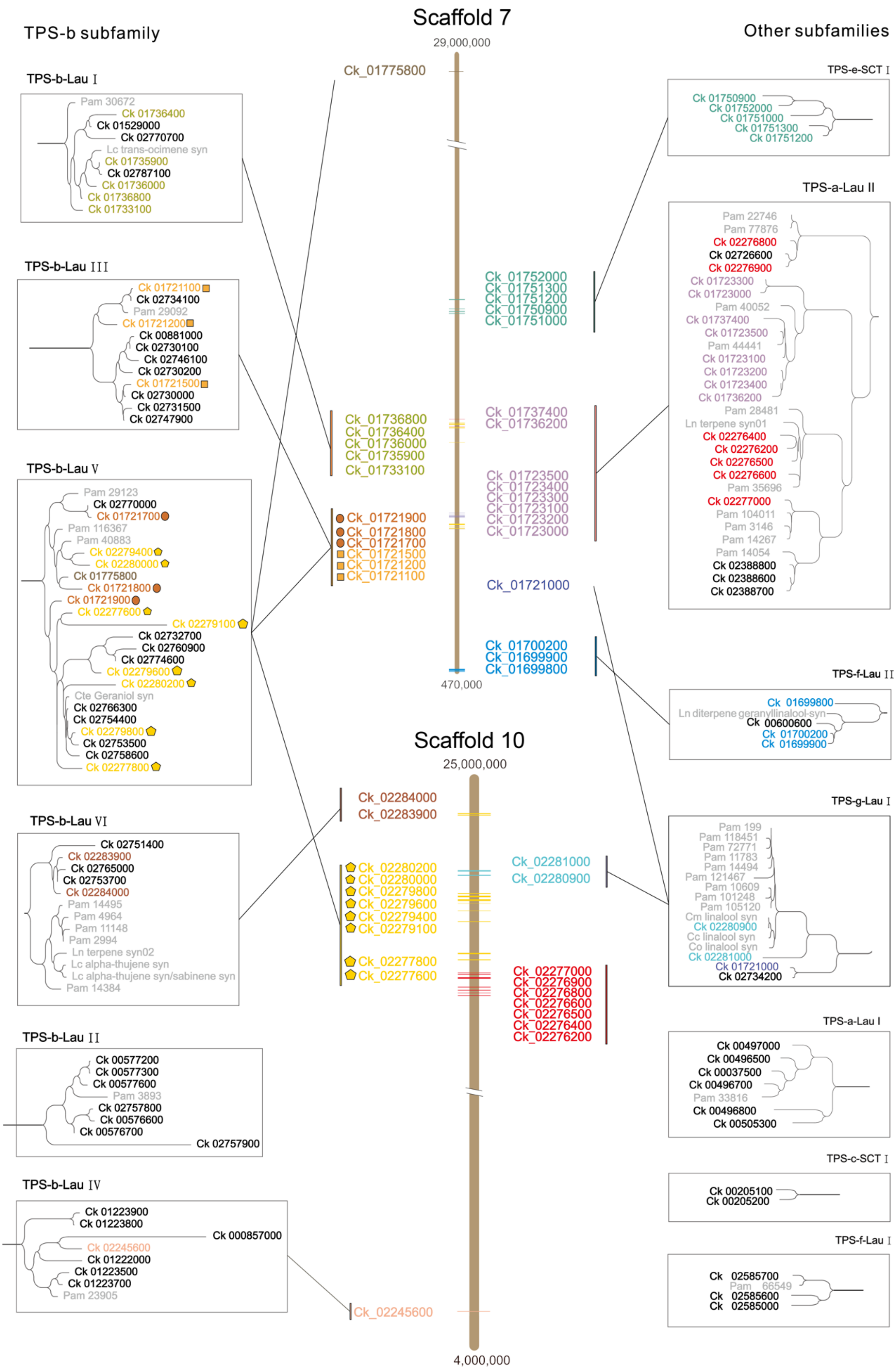

TPS-b subfamily
Scaffold 7
29,000,000
470,000
Other subfamilies
TPS-b-Lau Ⅰ
Pam 30672
Ck 01736400
Ck 01529000
Ck 02770700
Lc trans-ocimene syn
Ck 01735900
Ck 02787100
Ck 01736000
Ck 01736800
Ck 01733100
TPS-b-Lau Ⅲ
Ck 01721100
Ck 02734100
Pam 29092
Ck 01721200
Ck 00881000
Ck 02730100
Ck 02746100
Ck 02730200
Ck 01721500
Ck 02730000
Ck 02731500
Ck 02747900
TPS-b-Lau Ⅴ
Pam 29123
Ck 02770000
Ck 01721700
Pam 116367
Pam 40883
Ck 02279400
Ck 02280000
Ck 01775800
Ck 01721800
Ck 01721900
Ck 02277600
Ck 02279100
Ck 02732700
Ck 02760900
Ck 02774600
Ck 02279600
Ck 02280200
Cte Geraniol syn
Ck 02766300
Ck 02754400
Ck 02279800
Ck 02753500
Ck 02758600
Ck 02277800
TPS-b-Lau Ⅵ
Ck 02751400
Ck 02283900
Ck 02765000
Ck 02753700
Ck 02284000
Pam 14495
Pam 4964
Pam 11148
Pam 2994
Ln terpene syn02
Lc alpha-thujene syn
Lc alpha-thujene syn/sabinene syn
Pam 14384
TPS-b-Lau Ⅱ
Ck 00577200
Ck 00577300
Ck 00577600
Pam 3893
Ck 02757800
Ck 00576600
Ck 00576700
Ck 02757900
TPS-b-Lau Ⅳ
Ck 01223900
Ck 01223800
Ck 000857000
Ck 02245600
Ck 01222000
Ck 01223500
Ck 01223700
Pam 23905
Ck_01775800
Ck_01736800
Ck_01736400
Ck_01736000
Ck_01735900
Ck_01733100
Ck_01721900
Ck_01721800
Ck_01721700
Ck_01721500
Ck_01721200
Ck_01721100
Ck_01752000
Ck_01751300
Ck_01751200
Ck_01750900
Ck_01751000
Ck_01737400
Ck_01736200
Ck_01723500
Ck_01723400
Ck_01723300
Ck_01723100
Ck_01723200
Ck_01723000
Ck_01721000
Ck_01700200
Ck_01699900
Ck_01699800
Scaffold 10
25,000,000
4,000,000
Ck_02284000
Ck_02283900
Ck_02280200
Ck_02280000
Ck_02279800
Ck_02279600
Ck_02279400
Ck_02279100
Ck_02277800
Ck_02277600
Ck_02281000
Ck_02280900
Ck_02277000
Ck_02276900
Ck_02276800
Ck_02276500
Ck_02276600
Ck_02276400
Ck_02276200
Ck_02245600
TPS-e-SCT Ⅰ
Ck 01750900
Ck 01752000
Ck 01751000
Ck 01751300
Ck 01751200
TPS-a-Lau II
Pam 22746
Pam 77876
Ck 02276800
Ck 02726600
Ck 02276900
Ck 01723300
Ck 01723000
Pam 40052
Ck 01737400
Ck 01723500
Pam 44441
Ck 01723100
Ck 01723200
Ck 01723400
Ck 01736200
Pam 28481
Ln terpene syn01
Ck 02276400
Ck 02276200
Ck 02276500
Ck 02276600
Pam 35696
Ck 02277000
Pam 104011
Pam 3146
Pam 14267
Pam 14054
Ck 02388800
Ck 02388600
Ck 02388700
TPS-f-Lau Ⅱ
Ck 01699800
Ln diterpene geranyllinalool-syn
Ck 00600600
Ck 01700200
Ck 01699900
TPS-g-Lau Ⅰ
Pam 199
Pam 118451
Pam 72771
Pam 11783
Pam 14494
Pam 121467
Pam 10609
Pam 101248
Pam 105120
Cm linalool syn
Ck 02280900
Cc linalool syn
Co linalool syn
Ck 02281000
Ck 01721000
Ck 02734200
TPS-a-Lau I
Ck 00497000
Ck 00496500
Ck 00037500
Ck 00496700
Pam 33816
Ck 00496800
Ck 00505300
TPS-c-SCT Ⅰ
Ck 00205100
Ck 00205200
TPS-f-Lau Ⅰ
Ck 02585700
Pam 66549
Ck 02585600
Ck 02585000

Supplementary Fig. 29. Chromosome localization of CkTPS genes on scaffolds 7 and 10. The horizontal bars coded with colors correspond to TPS gene subfamilies in Supplementary Table 12. Phylogenetic trees within boxes (at the both sides of the scaffolds) correspond to Lauraceae-specific or SCT-specific subclades in the trees of each TPS subfamily (Supplementary Fig. 23–28. Genes on each partial tree in boxes are connected by lines to their locations in scaffolds). For visualization purposes, genes from the same sub-family were labeled in same color and icons (circle, square, and pentagon). CkTPS genes that located at other scaffolds and non-CkTPS genes were labeled in black and gray, respectively.

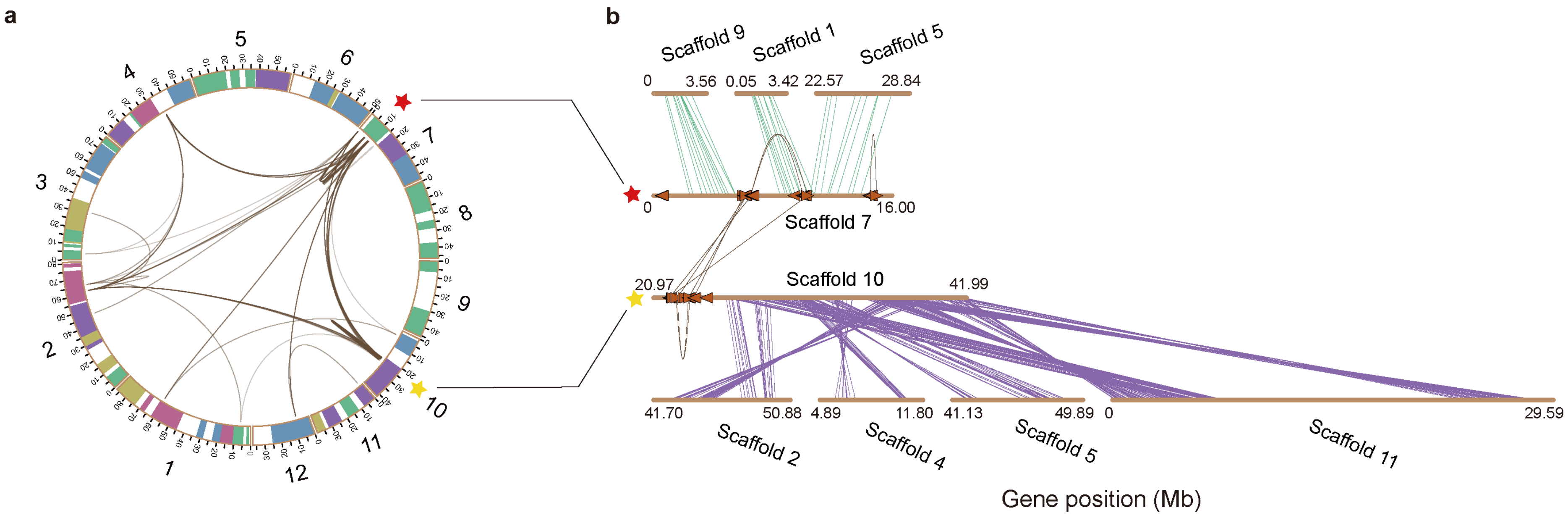

Supplementary Fig. 30. Chromosome localization of CkTPS genes on the largest 12 scaffolds. a, A circos plot showing distribution of CkTPS with links denoting CkTPS of different subfamilies. Numbers denote different scaffolds. Clustering of CkTPS on scaffold 7 and 10 were apparent. **b,** Schematic representation of intragenomic relationship amongst the synteny blocks around the CkTPS gene clusters. Lines denote orthologs defined by DAGchainer[1] and different colors denote assigned linkage groups.

III. SUPPLEMENTARY TABLES

Supplementary Table 1. Genome summary of *C. kanehirae* and 12 other plant species.

	A. trichopoda	*A. coerulea*	*A. thaliana*	***C. kanehirae***	*D. carota*	*G. biloba*	*M. acuminata*	*M. guttatus*	*O. sativa*
Genome size (Mb)	706.3	306.5	119.7	**730.7**	421.5	10,608.7	473.0	312.7	374.5
Scaffold number (n)	5,745	1,034	7	**2,153**	4,826	12	12	1,507	14
N50 (Mb)	4.9	43.6	23.5	**50.4**	36.6	1.4	34.1	21.2	30.0
Number of genes	27,313	30,023	27,416	**27,899**	32,113	36,528	36,528	28,140	42,189
Gene length (Mb)	153.1	107.5	60.5	**235.6**	100.1	1,122.5	138.9	77.1	121.1
Exon number	110,895	139,263	140,303	**150,741**	160,787	197,588	197,588	139,382	177,615
Exon length (Mb)	25.7	33.9	33.1	**36.4**	38.0	49.5	37.7	33.3	46.6
Intron length (Mb)	127.4	73.6	27.3	**199.2**	62.1	1,073.0	101.2	43.8	74.5
Intergene length (Mb)	553.2	199.1	59.2	**494.8**	321.4	9,486.2	334.0	235.7	253.4
BUSCO (%)	85.2	95.8	99.3	**88.5**	83.2	62.8	86.7	94.5	95.6

	P. abies	*P. trichocarpa*	*V. vinifera*	*Z. mays*
Genome size (Mb)	12,301.4	473.2	434.1	2,067.9
Scaffold number (n)	10,253,694	1,446	33	523
N50 (Mb)	0.0	17.4	19.5	23.0
Number of genes	70,736	41,335	26,346	63,480
Gene length (Mb)	171.7	107.7	128.4	170.0
Exon number	178,049	196,772	156,765	224,101
Exon length (Mb)	51.1	36.8	47.1	29.8
Intron length (Mb)	120.6	70.9	81.4	140.2
Intergene length (Mb)	12,129.6	365.6	305.7	1,897.9
BUSCO (%)	38.6	97.6	90.0	92.2

Supplementary Table 2. Summary of transcriptome dataset

Sample origin	Stage type[1]	Read length (bp)	Library size (bp)	num. reads	Accession
Flower buds	1	90	4,933,823,400	54,820,260	SRR7416917
Immature leaf	2	90	5,090,668,920	56,562,988	SRR7416906
Flower buds	3	90	5,395,165,920	59,946,288	SRR7416909
Old leaf	4	90	5313249000	59,036,100	SRR7416908
Young leaf	5	90	3,084,625,440	34,273,616	SRR7416918
Young stem	6	90	4,511,830,680	50,131,452	SRR7416905
Flowers	7	90	4,764,666,420	52,940,738	SRR7416910
Fruits	8	90	4,356,926,640	48,410,296	SRR7416911

[1]Photos of the stages are given in Supplementary Fig. 1c.

Supplementary Table 3. Statistics of orthologous group (OG) inferred by Orthofinder

Supplementary Table 3 is an Excel file.

Supplementary Table 4. Enriched gene ontology (GO) terms of genes located in region of heterozygousity (ROH)

Supplementary Table 4 is an Excel file.

Supplementary Table 5. Repeat content

Repeat classes	Genome proportion
LINE	5.04%
SINE	0.64%
LTR retrotransposons	25.53%
DNA transposons	12.67%
Unclassified	3.99%
Total	47.87%

LTR types	Proportion
Ty3/Gypsy	40.75%
Ty1/Copia	23.88%
Other	35.37%

Supplementary Table 6. Telomere scaffolds

Scaffold name	Scaffold length	Repeat start	Repeat end	Number of copies	Length from end of scaffold
Scaffold 1	87,013,940	86,974,270	86,992,249	2,547	21,691
Scaffold 8	46,631,104	46,616,307	46,628,952	1,801	2,152
Scaffold 13	4,922,974	4,772,062	4,792,208	2,821	130,766
Scaffold 867	17,896	1,445	17,896	2,355	0
Scaffold 901	17,187	11,108	17,187	868	0
Scaffold 1469	9,273	5,178	7,433	320	1,840

Supplementary Table 7. Distribution of NUPT lengths identified from the SCT nuclear genome

Scaffold no.	Length (bp)
**1**	85,208
**2**	106,958
**3**	78,893
**4**	61,608
**5**	67,435
**6**	55,275
**7**	43,295
**8**	43,134
**9**	37,243
**10**	37,481
**11**	32,187
**12**	38,239
**Total**	686,956
**Mean**	57,246

Supplementary Table 8. Distribution of NUPT lengths identified from the SCT nuclear genome

NUPT length (bp)	No. of NUPT	Cumulative %	Sum of NUPT length (bp)
**> 5000**	1	100	20,628
**3500–5000**	1	99.97	4,329
**2000–3499**	2	99.94	5,116
**1000–1999**	21	99.88	27,100
**500–999**	96	99.26	59,990
**250–499**	674	96.43	222,391
**< 250**	2,593	76.53	347,402
**Total**	3,388		686,956

Supplementary Table 9. Increased or reduced protein family domains (Pfam) in SCT

Supplementary Table 9 is an Excel file.

Supplementary Table 10. Enriched gene ontology (GO) terms of SCT's expanded gene families

Supplementary Table 10 is an Excel file. Enrichment was calculated by TopGO[7]

Supplementary Table 11. Enriched gene ontology (GO) terms of SCT's contracted gene families

Supplementary Table 11 is an Excel file. Enrichment was calculated by TopGO[7]

Supplementary Table 12. ***CkTPS*** **organization of six TPS subfamilies**

	TPS-a	TPS-b	TPS-c	TPS-e	TPS-f	TPS-g	Total
**Scaffold 1**	1	0	2	0	0	0	3
**Scaffold 2**	5	5	0	0	1	0	11
**Scaffold 3**	0	2	0	0	0	0	2
**Scaffold 4**	0	5	0	0	0	0	5
**Scaffold 5**	0	0	0	0	0	0	0
**Scaffold 6**	0	1	0	0	0	0	1
**Scaffold 7**	**8**	**12**	**0**	**5**	**3**	**1**	**29**
**Scaffold 8**	0	0	0	0	0	0	0
**Scaffold 9**	0	0	0	0	0	0	0
**Scaffold 10**	**7**	**11**	**0**	**0**	**0**	**2**	**19**
**Scaffold 11**	3	3	0	0	0	0	3
**Scaffold 12**	0	0	0	0	3	0	3
**Scaffold others**	1	22	0	0	0	1	24
**Total**	25	58	2	5	7	4	101

Supplementary Table 13. Examination of positive selection on the branches leading to the two TPS-f Lau clades using the branch-site model test[1].

Branch[2]	Model	-lnL	2δ(lnL)	*P* value	ω value	Positively selected sites[3]
TPS-f-Lau I	Null	32227.431			ω0 = 0.199, ω1 = 1, ω2 = 1	
	Alternative	32216.050	22.762	0.00001	ω0 = 0.201, ω1 = 1, ω2 = 894.197	403 W (0.959)
TPS-f-Lau II	Null	32232.241			ω0 = 0.205, ω1 = 1, ω2 = 1	
	Alternative	32228.503	7.476	0.006253	ω0 = 0.204, ω1 = 1, ω2 = 998.994	None

[1]This analysis was based on the tree topology of Supplementary Fig. 24. We used the Codeml program of PAML[8] to compare the null model (model = 2, Nsites =2, fixed omega = 1, omega =1) with the alternative model (model = 2, Nsites = 2, fixed omega = 0, omega = 1) in either four members of TPS-f Lau I clade or five members of TPS-f Lau II clade against all members plus the 12 non-magnoliids sequences (n=21). Four categories of sites were assumed[9]. They were (1) sites under purifying selection (ω0 < 1) on both foreground and background branches, (2) sites under neutral selection (ω0 = 1) on both foreground and background branches, (3) sites under positive selection (ω2 > 1) on the foreground branch and under purifying selection (ω0 < 1) on background branches, and (4) sites under positive selection (ω2 > 1) on the foreground branch and under neutral evolution (ω1 = 1) on background branches. In the null model, ω2 was fixed at 1.

[2]The examined branches are denoted by a circle in Supplementary Fig. 27.

[3]The position in the multiple sequence alignment and its amino acid residue are shown. The value inside the parenthesis is the posterior probability under Bayes empirical Bayes analysis.

References

1 Haas, B. J., Delcher, A. L., Wortman, J. R. & Salzberg, S. L. DAGchainer: a tool for mining segmental genome duplications and synteny. *Bioinformatics (Oxford, England)* **20**, 3643-3646, doi:10.1093/bioinformatics/bth397 (2004).

2 Quinlan, A. R. & Hall, I. M. BEDTools: a flexible suite of utilities for comparing genomic features. *Bioinformatics (Oxford, England)* **26**, 841-842, doi:10.1093/bioinformatics/btq033 (2010).

3 Vurture, G. W. *et al.* GenomeScope: fast reference-free genome profiling from short reads. *Bioinformatics* **33**, 2202-2204, doi:10.1093/bioinformatics/btx153 (2017).

4 Mirarab, S. & Warnow, T. ASTRAL-II: coalescent-based species tree estimation with many hundreds of taxa and thousands of genes. *Bioinformatics* **31**, i44-52, doi:10.1093/bioinformatics/btv234 (2015).

5 Matasci, N. *et al.* Data access for the 1,000 Plants (1KP) project. *Gigascience* **3**, 17, doi:10.1186/2047-217X-3-17 (2014).

6 Stamatakis, A. RAxML-VI-HPC: maximum likelihood-based phylogenetic analyses with thousands of taxa and mixed models. *Bioinformatics (Oxford, England)* **22**, 2688-2690, doi:10.1093/bioinformatics/btl446 (2006).

7 A, A. & J, R. topGO: Enrichment Analysis for Gene Ontology. *R package version 2.26.0* (2016).

8 Yang, Z. PAML 4: phylogenetic analysis by maximum likelihood. *Mol Biol Evol* **24**, 1586-1591, doi:10.1093/molbev/msm088 (2007).

9 Zhang, J., Nielsen, R. & Yang, Z. Evaluation of an improved branch-site likelihood method for detecting positive selection at the molecular level. *Mol Biol Evol* **22**, 2472-2479, doi:10.1093/molbev/msi237 (2005).
