# Supplementary material for: Stout camphor tree genome fills gaps in understanding of flowering plant genome evolution
Source: Nat Plants. 2019 Jan 9;5(1):63–73. doi: 10.1038/s41477-018-0337-0 (PMC6784883; doi:10.1038/s41477-018-0337-0)
Supplement: Supplementary file 2 — Reporting Summary [file 41477_2018_337_MOESM2_ESM.pdf]

nature research

Corresponding author(s): Shu-Miaw Chaw and Isheng Jason Tsai

Reporting Summary

Nature Research wishes to improve the reproducibility of the work that we publish. This form provides structure for consistency and transparency in reporting. For further information on Nature Research policies, see Authors & Referees and the Editorial Policy Checklist.

Statistical parameters

When statistical analyses are reported, confirm that the following items are present in the relevant location (e.g. figure legend, table legend, main text, or Methods section).

n/a	Confirmed	
☐	☒	The exact sample size (*n*) for each experimental group/condition, given as a discrete number and unit of measurement
☒	☐	An indication of whether measurements were taken from distinct samples or whether the same sample was measured repeatedly
☒	☐	The statistical test(s) used AND whether they are one- or two-sided<br>*Only common tests should be described solely by name; describe more complex techniques in the Methods section.*
☒	☐	A description of all covariates tested
☒	☐	A description of any assumptions or corrections, such as tests of normality and adjustment for multiple comparisons
☒	☐	A full description of the statistics including central tendency (e.g. means) or other basic estimates (e.g. regression coefficient) AND variation (e.g. standard deviation) or associated estimates of uncertainty (e.g. confidence intervals)
☒	☐	For null hypothesis testing, the test statistic (e.g. *F*, *t*, *r*) with confidence intervals, effect sizes, degrees of freedom and *P* value noted<br>*Give P values as exact values whenever suitable.*
☒	☐	For Bayesian analysis, information on the choice of priors and Markov chain Monte Carlo settings
☒	☐	For hierarchical and complex designs, identification of the appropriate level for tests and full reporting of outcomes
☒	☐	Estimates of effect sizes (e.g. Cohen's *d*, Pearson's *r*), indicating how they were calculated
☒	☐	Clearly defined error bars<br>*State explicitly what error bars represent (e.g. SD, SE, CI)*

Our web collection on statistics for biologists may be useful.

Software and code

Policy information about availability of computer code

Data collection	Pacbio reads were assembled using the FALCON (ver. 0.5.0) assembler. The consensus sequences of the assembly were further corrected using Pacbio reads using Quiver and Illumina reads using Pilon (ver. 1.22). The Pacbio assembly was scaffolded using HiRISE scaffolder (ver. July2015_GR) and consensus sequences were further improved using Pilon with one iteration. Genome prediction were performed using open-source tools : STAR (ver. 2.5.3a), Trinity (ver. 2.3.2), Stringtie (ver. 1.3.1c) , CLASS2 (ver. 2.1.7), GMAP (2017 -> 2018-07-04), MIKADO (ver 1.1). BRAKER1, MAKER2 (ver 2.31.9), Blast2GO and eggnog-mapper (ver 1.0.3).
Data analysis	R (3.3.4) was used for statistical analyses and figure generation. Commercial software were not used.

For manuscripts utilizing custom algorithms or software that are central to the research but not yet described in published literature, software must be made available to editors/reviewers upon request. We strongly encourage code deposition in a community repository (e.g. GitHub). See the Nature Research guidelines for submitting code & software for further information.

Data

Policy information about availability of data

All manuscripts must include a data availability statement. This statement should provide the following information, where applicable:

- Accession codes, unique identifiers, or web links for publicly available datasets
- A list of figures that have associated raw data
- A description of any restrictions on data availability

All of raw sequence reads used in this study have been deposited in NCBI under the BioProject accession number PRJNA477266. The assembly of SCT is available under the accession number SAMN09509728.

Field-specific reporting

Please select the best fit for your research. If you are not sure, read the appropriate sections before making your selection.

☒ Life sciences ☐ Behavioural & social sciences ☐ Ecological, evolutionary & environmental sciences

For a reference copy of the document with all sections, see nature.com/authors/policies/ReportingSummary-flat.pdf

Life sciences study design

All studies must disclose on these points even when the disclosure is negative.

Sample size	For comparative genome analyses, the amino acid and nucleotide sequences of 12 representative plant species were downloaded from various sources: Aquilegia coerulea, Arabidopsis thaliana, Daucus carota, Mimulus guttatus, Musa acuminata, Oryza sativa japonica, Populus trichocarpa, Vitis vinifera and Zea mays from Phytozome (ver. 12.1; https://phytozome.jgi.doe.gov/), Picea abies from the Plant Genome Integrative Explorer Resource (http://plantgenie.org/), Ginkgo biloba from GigaDB, and Amborella trichopoda from Ensembl plants (Release 39).
Data exclusions	No data exclusions in this manuscript
Replication	No replication in this manuscript
Randomization	No randomization in this manuscript as genomes assemblies were not allocated into experimental groups.
Blinding	No blinding in this manuscript as the data were not allocated into groups

Reporting for specific materials, systems and methods

Materials & experimental systems

n/a	Involved in the study
☒	☐ Unique biological materials
☒	☐ Antibodies
☒	☐ Eukaryotic cell lines
☒	☐ Palaeontology
☒	☐ Animals and other organisms
☒	☐ Human research participants

Methods

n/a	Involved in the study
☒	☐ ChIP-seq
☐	☒ Flow cytometry
☒	☐ MRI-based neuroimaging

Flow Cytometry

Plots

Confirm that:

☐ The axis labels state the marker and fluorochrome used (e.g. CD4-FITC).

☐ The axis scales are clearly visible. Include numbers along axes only for bottom left plot of group (a 'group' is an analysis of identical markers).

☐ All plots are contour plots with outliers or pseudocolor plots.

☒ A numerical value for number of cells or percentage (with statistics) is provided.

Methodology

Sample preparation	Tissues of about 0.25 square centimeters were excised from freshly collected leaves, and placed in plastic petri dishes (35 mm x 10 mm) on ice, and then sliced to fine pieces using a new razor blade in extraction buffer for 1~2 min.
Instrument	The data were collected using MoFlo XDP Cell Sorter (Beckman Coulter Life Science, Indianapolis, IN) and Attune NxT Flow Cytometer (Thermo Fisher Scientific Inc., Waltham, MA).
Software	Summit (ver5.3)
Cell population abundance	Flow cytometry was used for quantification purposely only, and no post-sorting fraction was collected.
Gating strategy	Filter-575-25 was used in gating. The FSC-Area/SSC-Area gate (R1) method was used to eliminate debris, cell fragments, and dead cells. Single cell and double cells were discriminated by using RPE-Height/ RPE-Area (R4).

☐ Tick this box to confirm that a figure exemplifying the gating strategy is provided in the Supplementary Information.
